# Tomato *Male sterile 10*^*35*^ is essential for pollen development and meiosis in anthers

**DOI:** 10.1093/jxb/eru389

**Published:** 2014-09-26

**Authors:** Hee-Jin Jeong, Jin-Ho Kang, Meiai Zhao, Jin-Kyung Kwon, Hak-Soon Choi, Jung Hwan Bae, Hyun-ah Lee, Young-Hee Joung, Doil Choi, Byoung-Cheorl Kang

**Affiliations:** ^1^Department of Plant Science and Plant Genomics and Breeding Institute, College of Agriculture and Life Science, Seoul National University, 599 Gwanak-ro Gwank-gu, Seoul 151-921, Republic of Korea; ^2^Plant Genomics and Breeding Institute, College of Agricultural Sciences, Seoul National University, 599 Gwanak-ro Gwank-gu, Seoul 151-921, Republic of Korea; ^3^College of Life Science, Qingdao Agricultural University, Qingdao 266-109, PR China; ^4^National Institute of Horticultural and Herbal Science, Suwon 440-310, Republic of Korea; ^5^School of Biological Sciences and Technology, Chonnam National University, Gwangju 500-757, Republic of Korea

**Keywords:** Anther, male sterility, meiosis, tapetum, tomato (*Solanum lycopersicum*).

## Abstract

Male fertility in flowering plants depends on proper cellular differentiation in anthers. Meiosis and tapetum development are particularly important processes in pollen production. In this study, we showed that the tomato *male sterile* (*ms10* ^*35*^) mutant of cultivated tomato (*Solanum lycopersicum*) exhibited dysfunctional meiosis and an abnormal tapetum during anther development, resulting in no pollen production. We demonstrated that *Ms10* ^*35*^ encodes a basic helix–loop–helix transcription factor that is specifically expressed in meiocyte and tapetal tissue from pre-meiotic to tetrad stages. Transgenic expression of the *Ms10* ^*35*^ gene from its native promoter complemented the male sterility of the *ms10* ^*35*^ mutant. In addition, RNA-sequencing-based transcriptome analysis revealed that *Ms10* ^*35*^ regulates 246 genes involved in anther development processes such as meiosis, tapetum development, cell-wall degradation, pollen wall formation, transport, and lipid metabolism. Our results indicate that *Ms10* ^*35*^ plays key roles in regulating both meiosis and programmed cell death of the tapetum during microsporogenesis.

## Summary

This study demonstrated that tomato *Male sterile 10* ^*35*^ encodes a basic helix–loop–helix transcription factor involved in meiosis and tapetum development at the early stage of anther development.

## Introduction

Pollen development is one of the most fundamental processes in the plant life cycle (Wilson and Zhang, 2009). Through pollen, plants deliver genetic material and expand genetic diversity by producing recombinant progeny in the subsequent generation (Deveshwar *et al.*, 2011). Pollen development involves an exquisite pathway supported by cellular changes and the regulation of an enormous number of genes (Honys and Twell, 2004; Wilson and Zhang, 2009; Feng *et al.*, 2012).

In *Arabidopsis* (*Arabidopsis thaliana*) and rice (*Oryza sativa*), anther development has been well studied and many of the genes involved have been identified. In *Arabidopsis*, after the floral structures are successively generated, anther cells initiate specification and differentiation to form a bilaterally symmetrical structure with four lobes. Archesporial cells in each lobe generate five distinct cell layers (from outer to inner: epidermis, endothecium, middle layer, tapetum, and the sporogenous cell) (Smyth *et al.*, 1990; Wilson and Zhang, 2009). At the meiotic stage, meiocytes (pollen mother cells), which are developed from sporogenous cells, undergo meiotic cell divisions and are separated from the tapetal cell layer. Tapetal cells become vacuolated and initiate programmed cell death (PCD)-triggered degradation. During meiosis I, homologous chromosomes replicate, pair, synapse, and undergo recombination by exchanging DNA (Zickler and Kleckner, 1999). After that, homologous chromosomes are aligned and pulled towards opposite poles by spindle organization. Finally, dyads are produced (Ma, 2005). For example, in prophase I, rice *HOMOLOGOUS PAIRING ABERRATION IN RICE MEIOSIS 1* (*PAIR1*), *PAIR2*, and *PAIR3* are important for chromosome pairing and synapsis, respectively (Nonomura *et al.*, 2004, 2006; Yuan *et al.*, 2009). *Arabidopsis* *SWITCH1* (*SWI1*) and rice *MEIOTIC RECOMBINATION PROTEIN8* (*REC8*) are essential for chromatid cohesion and bivalent formation (Mercier *et al.*, 2003; Shao *et al.*, 2011). In metaphase I, *Arabidopsis MULTIPOLAR SPINDLE1* (*MPS1*) plays a role in organization of the spindle and chromosomal segregation (Jiang *et al.*, 2009). In anaphase, rice *POLLEN SEMI-STERILITY1* (*PSS1*), which encodes a kinesis-1 like protein, is necessary for meiotic chromosome pulling by the spindle (Zhou *et al.*, 2001). Mutations of these genes related to meiosis cause defective meiocyte development and male sterility.

During meiosis, tapetal cells surrounding meiocytes produce various enzymes, lipids, starch, pollen wall materials, and other molecules required for pollen development (Goldberg *et al.*, 1993; Zhang *et al.*, 2006). For instance, *Arabidopsis EXTRA SPOROGENOUS CELLS/EXCESS MICROSPOROCYTES1* (*EMS1*/*EXS*) and *TAPETAL DETERMINANT1* (*TPD1*) are important for tapetal specification and maintenance of tapetal cell fate, and these mutants show extra meiocytes and no tapetal cells (Canales *et al.*, 2002; Yang *et al.*, 2003; Zhao *et al.*, 2002). Mutations in *Arabidopsis DYSFUNCTIONAL TAPETUM1* (*DYT1*) and rice *UNDEVELOPED TAPETUM1* (*UDT1*) cause abnormal tapetal development such as vacuolated tapetal cells, resulting in male sterility (Jung *et al.*, 2005; Zhang *et al.*, 2006). In addition, *EMS1*/*EXS* and *TPD1* are also required for cytokinesis after chromosomal segregation (Canales *et al.*, 2002; Yang *et al.*, 2003; Zhao *et al.*, 2002), suggesting that sporophytic cells and gametophytic cells coordinate with each other by cell-to-cell communication.

Tomato flowers contain five sepals that alternate with five petals, in addition to five stamens and a style formed by two fused carpels. The stamens, which house pollen production, sit inside the petals. A single tomato stamen consists of two elongated compartments, and the individual stamens are fused together to form an anther cone called the androecium, which surrounds the style. In the cultivated tomato, the stigma is completely covered under the staminal tube (Brukhin *et al.*, 2003). Tomato pollen development is quite similar to that of *Arabidopsis* and rice (Brukhin *et al.*, 2003, Wilson and Zhang, 2009; D. Zhang *et al.*, 2011). Tomato stamen primordia are initiated at the early stage of anther development followed by archesporial cell differentiation. Sporogenous and parietal cells are differentiated from archesporial cells. These cells give rise to microspore tetrads and tapetum, respectively, after going through meiosis. Finally, the microspores mature and become pollen grains (Rasmussen and Green, 1993; Brukhin *et al.*, 2003). In tomato, over 50 male-sterile mutants have been reported, and they can be divided into three classes (functional, structural, and sporogenous) based on their developmental defects (Gorman and McCormick, 1997). Sporogenous male-sterile mutants can be further classified into five groups (pre-meiotic, meiotic, tetrad, microspore, and not determined) according to the stage at which pollen development aborts or breaks down (Rick and Butler, 1956; Gorman and McCormick, 1997). For example, the functional male-sterile mutant *positional sterile-2* (*ps-2*) is defective in pollen dehiscence. Pre-meiotic mutants such as *male sterile* (*ms*) *3* and *ms15* display pollen mother cell (PMC) collapse or abortion prior to the meiotic prophase. Meiotic mutants such as *ms5* and *ms10* ^*35*^ (allelic to *ms10*) show defects in tapetal tissue (Rick and Butler, 1956; Gorman and McCormick, 1997). While many tomato male-sterile mutants are available, the only known underlying gene was the *polygalacturonase* gene responsible for the *ps-2* male-sterile mutant (Gorguet *et al.*, 2009).

The *ms10* ^*35*^ mutant was described previously as a spontaneous mutant with defects in tapetum development and degeneration (Rick, 1948; Zamir *et al.*, 1980; Corral-Martínez *et al.*, 2011). Because of its stable male sterility and lack of growth defects, it has been widely used for F_1_ hybrid breeding (Georgiev, 1991; Kumar and Singh, 2005). In addition, the *ms10* ^*35*^ mutant also has been used for anther culture to generate haploid plants, because a callus can easily be induced from its anthers (Zamir *et al.*, 1980; Corral-Martínez *et al.*, 2011). Here, we demonstrated that the *ms10* ^*35*^ mutant is defective in chromosome segregation at anaphase I during meiosis, as well as in tapetum development, causing male sterility. Using a map-based cloning approach, we found that *Ms10* ^*35*^ encodes a basic helix–loop–helix (bHLH) transcription factor. RNA sequencing (RNA-seq)-based transcriptome analysis revealed that *Ms10* ^*35*^ regulates 246 genes related to meiosis, tapetum development, lipid metabolism, cell wall modification/degradation, and pollen wall biosynthesis. These results demonstrated that *Ms10* ^*35*^ serves as a master regulator of pollen development in tomato.

## Materials and methods

### Plant material and plant growth

A tomato male-fertile parent (T-1082) and male-sterile *ms10* ^*35*^ (2–517), which was backcrossed to T-1082 six times, were obtained from the National Institute of Horticultural and Herbal Science (Suwon, Korea). T-1082 and the backcrossed *ms10* ^*35*^ were used in all experiments except the mapping experiment in which the original *ms10* ^*35*^ was crossed to T-1082. Seedlings were grown in 50-plug trays containing sterilized soil in a growth chamber maintained under 18h light (265 mE m^–2^ s^–1^) at 27 °C and 6h darkness at 18 °C and 60% humidity. At the eight-leaf stage, the seedlings were transplanted to a greenhouse in the farm of the College of Agriculture and Life Science at Seoul National University (Suwon, Korea).

### Microscopy

Fluorescein diacetate (FDA) was used to check pollen viability according to the protocol of Kim *et al.* (2013). FDA-stained pollen was examined using an Axiophot microscope (Zeiss, Oberkochen, Germany). For ultrastructure and transmission electron microscopy (TEM) analysis, floral buds were infiltrated with Spurr’s resin according to the protocol of Kim *et al.* (2013). TEM images were observed using a JEM1010 transmission electron microscope (Jeol, Tokyo, Japan) at 80kV. For scanning electron microscopy (SEM), pollen grains of mature flowers were mounted and coated with palladium-gold in a sputter coater (BAL-TEC/SCD 005; Balzers, Lichtenstein) and examined using a field emission scanning electron microscope (SUPRA 55VP; Carl Zeiss, Germany) with an acceleration voltage of 15kV.

### 4′,6-Diamidino-2-phenylindole (DAPI) staining analysis of meiotic processes

For the observation of meiotic chromosomes, a modified PMC spreading protocol was used (Kwon and Kim, 2009). Briefly, floral buds around the meiotic stage were fixed in Carnoy’s fixative solution (ethanol:acetic acid=3:1, v/v) for 48h. Fixed buds were rinsed twice in distilled water and then once in 10mM citrate buffer (pH 4.5). Samples were incubated at 37 °C for 3h in a digestion mix containing 2% (w/v) cellulase RS, 1% (w/v) pectinase, and 0.5% (w/v) pectolyase Y23 (Sigma, St Louis, MO, USA) dissolved in 10mM citrate buffer. After digestion, cells were fixed in 60% acetic acid on a heated slide. After air drying, fixed cells were stained and mounted with 2 μg ml^–1^ of DAPI solution in Vectashield anti-fade mounting medium (Vector Laboratories, Burlingame, CA, USA).

### DNA extraction

Genomic DNA was extracted from two to three young leaves using a hexadecyltrimethyl-ammonium bromide method (Jeong *et al.*, 2010). Leaf tissue was fragmented using TissueLyserII (Qiagen, Haan, Germany). DNA concentrations were measured with a Nanodrop spectrophotometer (NanoDrop Technologies, Wilmington, DE, USA) and diluted to a final concentration of 20ng μl^–1^ in TE buffer (pH 7.0) for further experiments.

### Bacterial artificial chromosome (BAC) alignment and *Ms10* ^*35*^-linked marker development

The *Ms10* ^*35*^ gene is known to be located between the *PEROXIDASE-2* (*PRX-2*) and *ANTHOCYANIN ABSENT* (*AA*) genes, which are around the 69–78 cM region on chromosome 2 (Tanksley and Rick, 1980; Tanksley *et al.*, 1992). Tomato BAC clones corresponding to the 69–78 cM region were aligned and assembled by Seqman software (DNA Star; DNASTAR, Madison, WI, USA). In order to develop *Ms10* ^*35*^ *-*linked markers, primer sets were randomly designed within the 69–78 cM region from the assembled BAC clones. The designed primers were tested for polymorphism between parental DNA (*ms10* ^*35*^ and T-1082) and F_1_ DNA derived from a cross between *ms10* ^*35*^ and T-1082 plants using high-resolution-melting (HRM) analysis (Rotor-Gene 6000 thermocycler; Corbett Research, Sydney, Australia) according to a previously described method (Jeong *et al.* 2011).

### Genetic analysis and map-based cloning of *Ms10* ^*35*^

Fine mapping of *Ms10* ^*35*^ was performed with an F_2_ population derived from a cross between *ms10* ^*35*^ mutant and T-1082 plants, and was facilitated by the assembled BAC sequence described above and the tomato genome sequence (Tomato Genome Consortium, 2012). A population of 1100 F_2_ plants was scored for male sterility and subsequently genotyped with HRM markers (Supplementary Table S1 at *JXB* online). Linkage analysis of molecular markers was conducted using the Carthagene 1.0 program (de Givry *et al.*, 2005). *Ms10* ^*35*^ was positioned to an ~80kb region on chromosome 2 flanked by markers 762K and 843K. Putative genes in the 80kb region were predicted using the FGENESH program (http://linux1.softberry.com/), the tomato Unigene database from SGN (http://solgenomics.net), and the BLASTP interface of the National Center for Biotechnology Information (http://www.ncbi.nlm.nih.gov/).

### Total RNA isolation and reverse transcription (RT)-PCR

Floral buds at different stages, leaves, stems, and fruits were collected from *ms10* ^*35*^ and T-1082 plants and quickly frozen in liquid nitrogen. Total RNA was isolated using Trizol extraction buffer (Ambion, Carlsbad, CA, USA) according to the manufacturer’s protocol. cDNA was synthesized from 2 μg of total RNA using reverse transcriptase (Promega, Madison, WI, USA). cDNA (200ng) was used for RT-PCR. Amplified PCR products were separated on a 1% agarose gel and stained with ethidium bromide. The primer sequences used for RT-PCR are listed in Supplementary Table S1.

### Rapid amplification of cDNA ends (RACE)

To identify the transcription start site of the *Ms10* ^*35*^ gene, 5ʹRACE-PCR was performed using a SMARTer™ RACE cDNA Amplification kit (Clontech Laboratories, Mountain View, CA, USA). RNA was extracted from T-1082 anthers and cDNA was synthesized according to the manufacturer’s instructions. Sequencing analysis was performed at the National Instrumentation Center for Environmental Management (NICEM, Seoul National University, Seoul, Korea).

### Genome-walking PCR

To identify the mutated region of the *ms10* ^*35*^ gene, genome-walking PCR was performed using a Genome Walker kit (Clontech Laboratories, Mountain view, CA, USA) according to the manufacturer’s manual. Sequence information for gene-specific primers 1 and 2 is provided in Supplementary Table S1. The fragment amplified by genome-walking PCR was cloned into a pGEM-T vector (pGEM^®^-T Easy Vector Systems, Promega, Seoul, Korea) and sequenced at the NICEM.

### Complementation of the *ms10* ^*35*^ mutation

A 2992bp genomic sequence of *Ms10* ^*35*^, which contained the entire coding region (1002bp) with 1.4kb of upstream sequence and 0.6kb of downstream sequence, was amplified with *Xba*I restriction site-tagged primers (Supplementary Table S1). The resulting fragment was digested with *Xba*I, purified, and cloned into the *Xba*I site of the pCAMBIA2300 binary vector to generate pCAMBIA2300::*Ms10* ^*35*^. The resulting construct and pCAMBIA2300 were introduced into *Agrobacterium tumefaciens* strain LBA4404 and used to transform *ms10* ^*35*^ heterozygous (*Ms10* ^*35*^/*ms10* ^*35*^) cotyledon explants, as described previously (Seong *et al.*, 2007). The presence of the T-DNA insert in independent primary (T_0_) transformants was confirmed by PCR using a *NPTII*-specific primer set (Supplementary Table S1) to amplify a fragment of the *NPTII* gene. Subsequently, independent T_1_ plants with the homozygous *ms10* ^*35*^/*ms10* ^*35*^ genotype were selected using *ms10* ^*35*^-specific primers (Supplementary Table S1). From these, F_1_ plants having both *NPTII* and *Ms10* ^*35*^ transgenes were selected using *NPTII*-specific and *Ms10* ^*35*^ transgene-specific primers, respectively (Supplementary Table S1).

### RNA transcriptome analysis by RNA-seq

Total RNA from floral buds at stages 1 to 3 was extracted in the same manner as described above. A strand-specific RNA-seq library was constructed for the synthesis of cDNA as described by Zhong *et al.* (2011). RNA transcriptome was obtained using Hiseq 2500 (Illumina/Solexa, San Diego, CA, USA) at NICEM. The RNA-seq algorithm of the CLC Genomics Workbench 6.0 was used for relative digital expression with a 98% identity threshold (CLC bio, Prismet, Denmark). Digital expression data were normalized and transformed using CLC Genomics Workbench 6.0 internal algorithms. ITAG2.3_CDS was used as a reference genome for read mapping (http://solgenomics.net). The mean values of three biological replicates were transformed into log_2_ values (*ms10* ^*35*^/T-1082). The DESeq tool of the R package (http://www.bioconductor.org/) was used to identify differentially expressed genes in *ms10* ^*35*^ compared with T-1082 with a false discovery rate of <0.05 (Anders *et al.*, 2013).

## Results

### The *ms10* ^*35*^ mutant does not produce pollen

**Fig. 1.**
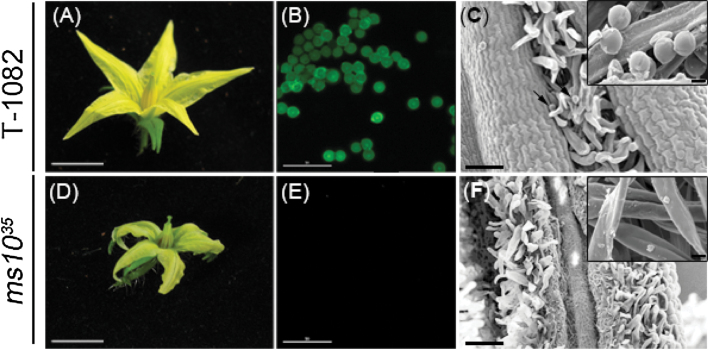
Flower phenotypes and pollen production in T-1082 (male fertile) and the *ms10* ^*35*^ mutant. (A, D) Flowers of T-1082 (A) and *ms10* ^*35*^ plants (D). (B, E) FDA assay for pollen viability of T-1082 (B) and *ms10* ^*35*^ (E) plants. Pollen grains or dust particles released from anthers at the dehiscence stage were used. (C, F) SEM observation of the surfaces of anthers at the dehiscence stage from T-1082 (C) and *ms10* ^*35*^ (F) plants. The insets show a closer look at the surface of the anthers. The T-1082 anther produced pollen grains (arrow and inset). Bars, 0.5cm (A, D); 100 µm (B, C, E, F); 10 μm (insets). (This figure is available in colour at *JXB* online.)

The tomato male-sterile mutant *ms10* ^*35*^ has been described as being defective in the production of active pollen (Rick, 1948; Zamir *et al.*, 1980). To examine developmental defects, *ms10* ^*35*^ plants were compared with male-fertile T-1082 plants. There was no difference in development between *ms10* ^*35*^ and T-1082 plants until the flowering stage. At the flowering stage, *ms10* ^*35*^ plants showed morphological differences in flower shape. The *ms10* ^*35*^ mutant had much smaller flowers compared with T-1082 plants (83% of T-1082, Fig. 1A, D, and Supplementary Fig. S1B at *JXB* online). In addition, the anther cone and the style in *ms10* ^*35*^ flowers were much shorter than in T-1082 flowers (60 and 86% of T-1082, Supplementary Fig. S1B). Due to the more dramatic change in anther cone length compared with style length, the styles of *ms10* ^*35*^ flowers protruded over the anther cones (Fig. 1D and Supplementary Fig. S1B). To check whether *ms10* ^*35*^ flowers produced viable pollen grains, an FDA assay, which measures cell viability, was performed with mature pollen grains or dust particles released from anthers at the dehiscence stage. The pollen of T-1082 flowers showed green fluorescence whereas no signals were detected from *ms10* ^*35*^ mutant flowers (Fig. 1B, E), indicating that *ms10* ^*35*^ flowers did not produce viable pollen. To confirm that *ms10* ^*35*^ flowers produced no pollen, we used SEM. T-1082 anthers contained normal globular pollen grains, but *ms10* ^*35*^ anthers had no pollen (Fig. 1C, F). These results demonstrated that the male sterility of the *ms10* ^*35*^ mutant resulted from a lack of production of pollen.

### Pollen development in *ms10* ^*35*^ is arrested at the tetrad stage

**Fig. 2.**
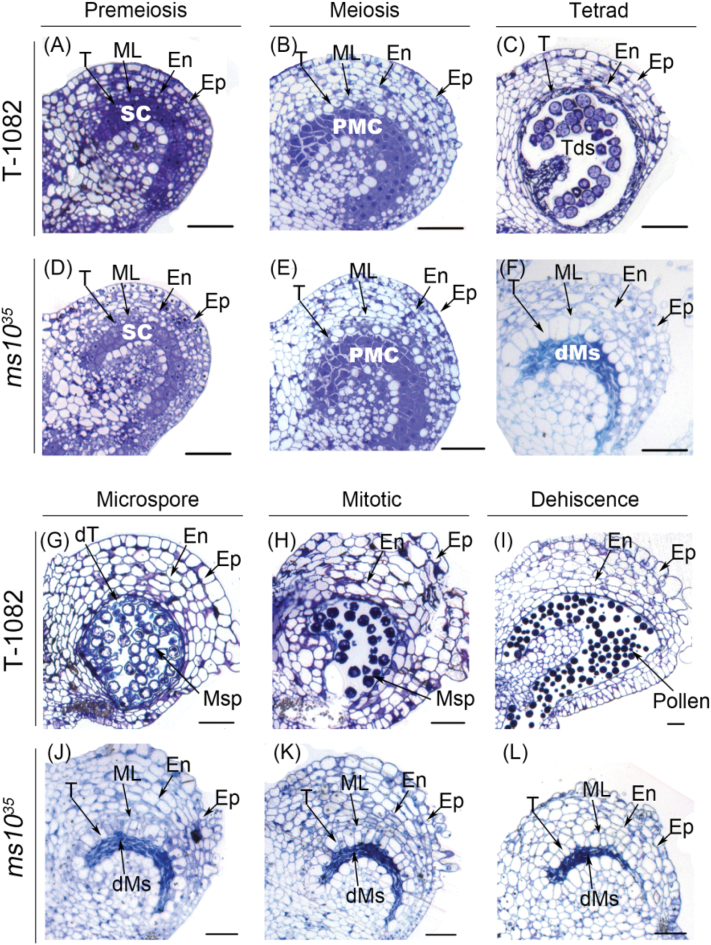
Histological characterization of anther development in T-1082 and *ms10* ^*35*^ flowers. Cross sections of T-1082 (A–C, G–I) and *ms10* ^*35*^ (D–F, J–L) anthers at pre-meiotic (A, D), meiotic (B, E), tetrad (C, F), microspore (G, J), mitotic (H, K), and dehiscence (I, L) stages. dMs, degenerated meiocytes; dT, degenerated tapetum; En, endothecium; Ep, epidermis; ML, middle cell layer; Msp, microspore; PMC, pollen mother cell; SC, sporogenous cell; T, tapetum; Tds, tetrads. Bars, 50 μm. (This figure is available in colour at *JXB* online.)

To determine the spatial and temporal occurrence of defects in *ms10* ^*35*^ anthers, we prepared thin sections from anthers at different stages of development and examined them using light microscopy. At the pre-meiotic and meiotic stages of T-1082 and *ms10* ^*35*^ anthers, the five different cell layers were successfully differentiated from archesporial cells (Fig. 2A, D), and sporogenous cells developed into PMCs and underwent meiosis (Fig. 2B, E). At the tetrad stage, however, dramatic morphological differences were observed between *ms10* ^*35*^ and T-1082 anthers. In T-1082 anthers, PMCs divided into tetrads after meiosis. Tapetal cells were greatly condensed and deeply stained. The middle cell layer was degenerated and almost invisible (Fig. 2C). In *ms10* ^*35*^ anthers, PMCs were crushed and failed to produce tetrads. Tapetal cells and the middle cell layer were excessively enlarged and vacuolated (Fig. 2F). At the microspore stage in T-1082 anthers, free microspores were released into anther locules (Fig. 2G). In *ms10* ^*35*^ anthers at the microspore stage, degenerated meiocytes were aggregated and gradually degraded. Vacuolated tapetal cells and middle cell layers were severely expanded (Fig. 2J). At the mitotic and dehiscence stages in T-1082 anthers, vacuolated microspores were deeply stained with toluidine blue due to the accumulation of collapsed tapetum fragments and nutrients, and the tapetum and middle cell layer had already disappeared (Fig. 2H). Finally, anthers dehisced and pollen grains were released (Fig. 2I). By contrast, in *ms10* ^*35*^ anthers at the mitotic and dehiscence stages, degenerated meiocytes continued to dwindle, while tapetal cells remained swollen and vacuolated without degeneration (Fig. 2K, L).

### The *ms10* ^*35*^ mutant is defective in tapetum development

**Fig. 3.**
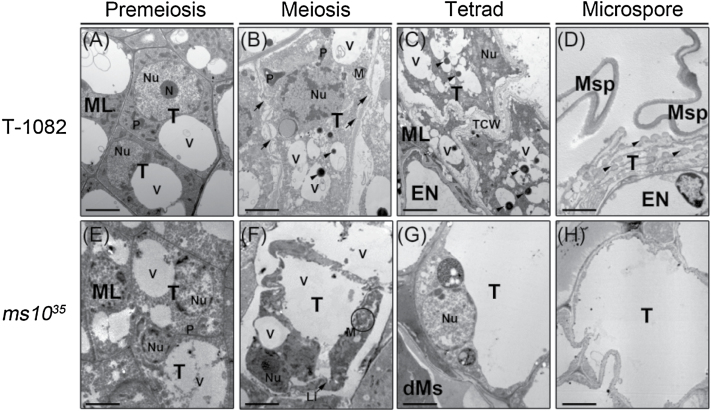
TEM analysis of T-1082 and *ms10* ^*35*^ tapetum. Tapetal cells of T-1082 (A–D) and *ms10* ^*35*^ (E–H) anthers at pre-meiotic (A, E), meiotic (B, F), tetrad (C, G), and microspore (D, H) stages. At the meiotic stage, T-1082 anthers (B) contained nuclei, plastids, mitochondria, and vacuoles. Arrowheads indicate electron-dense deposits in the vacuoles. Arrows indicate plasmodesmata. In *ms10* ^*35*^ anthers, the nuclei appeared normal, but larger vacuoles were generated. Mitochondria (indicated by a circle) and lipid structures were also detected (F). At the tetrad stage, T-1082 anthers (C) showed an irregular tapetum cell wall, and a degenerated nucleus and cellular organelles. Arrowheads indicate electron-dense deposits in the vacuoles. *ms10* ^*35*^ anthers (G) had extremely vacuolated tapetum and intact nuclei and organelles. At the microspore stage, T-1082 anthers (D) exhibited degenerated tapetal cells and production of orbicules (arrowheads). *ms10* ^*35*^ anthers (H) were completely vacuolated. dMs, degenerated meiocytes; EN, endothecium; Li, lipid deposit; M, mitochondria; ML, middle cell layer; Msp, microspore; N, nucleolus; Nu, nucleus; P, plastid; T, tapetum; TCW, tapetal cell wall; V, vacuole. Bars, 2 μm.

To examine in detail the defects of tapetal cells in the *ms10* ^*35*^ mutant, we used TEM. In agreement with the light microscopic observations, there was no noticeable difference between *ms10* ^*35*^ and T-1082 at the pre-meiotic stage (Fig. 3A, E). At the meiotic stage, tapetal cells in T-1082 anthers were still well defined, but more vacuoles were generated and the nuclear membrane began disappearing, indicating that PCD-triggered cell degradation had already commenced (Fig. 3B). By contrast, ms*10* ^*35*^ tapetal cells at the meiotic stage showed abnormal morphology: the cytoplasm of ms*10* ^*35*^ tapetal cells was highly vacuolated and showed extensive lipid deposits (Fig. 3F). At the tetrad stage in T-1082, the cytoplasm was condensed and deeply stained. The nuclear membrane and cellular organelles had disappeared. Electron-dense deposits were observed in the vacuoles (Fig. 3C). At the tetrad stage of *ms10* ^*35*^, tapetal cells were greatly expanded and vacuolated. Cellular organelles including the nucleus still maintained their structures (Fig. 3G). At the microspore stage of T-1082 tapetal cells, the cytoplasm and cell walls were diminished. Nuclei and cellular organelles were also completely absent. Instead, orbicules were distributed along the loosened tapetum cells facing the microspore (Fig. 3D). In *ms10* ^*35*^ tapetal cells at the microspore stage, tapetal cells were still enlarged and vacuolated (Fig. 3H). These observations reveal that the *ms10* ^*35*^ mutant had abnormally vacuolated tapetal cells with aborted degeneration.

### Development of PMCs in the *ms10* ^*35*^ mutant is arrested at anaphase I during meiosis

**Fig. 4.**
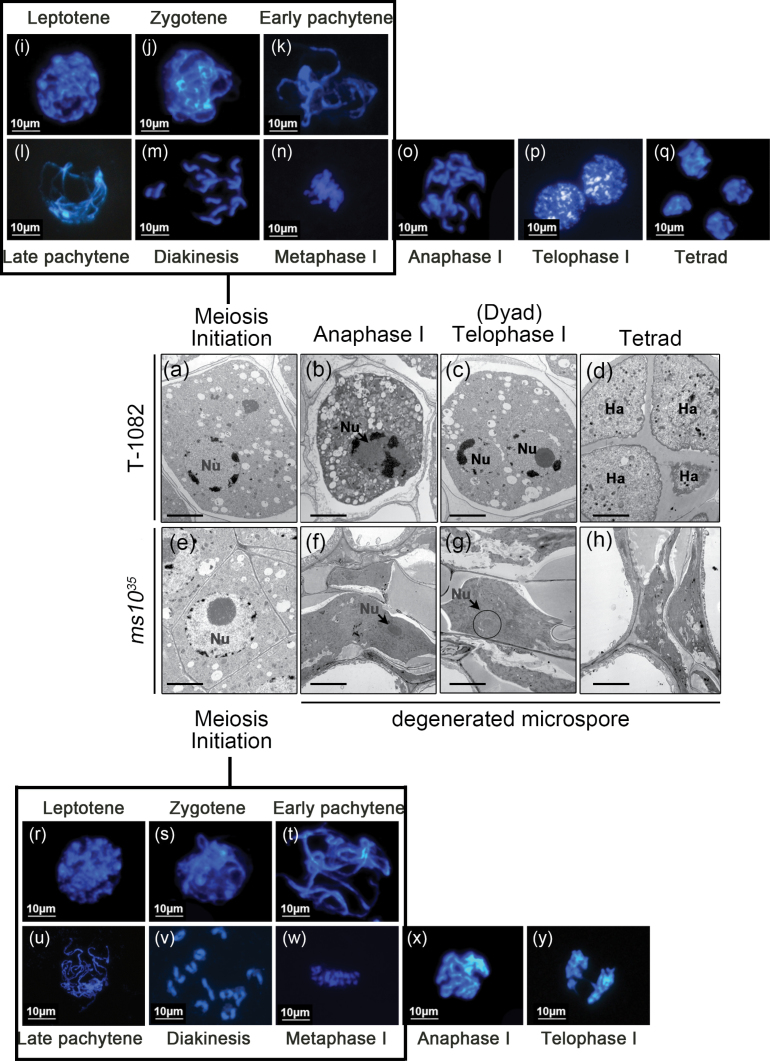
TEM analysis and DAPI staining for observation of meiosis in T-1082 and *ms10* ^*35*^. (A–H) TEM micrographs of meiocyte cells from T-1082 (A–D) and *ms10* ^*35*^ (E–H) anthers at meiosis initiation (A, E), anaphase I (B, F), telophase I (C, G), and tetrad (D, H) stages. At anaphase I, the meiocyte of T-1082 anthers was divided (arrow and white circles, B), while the meiocyte of *ms10* ^*35*^ anthers failed to divide and was diminished (arrow, F). At telophase I (dyad stage), the meiocyte of T-1082 anthers was divided into a dyad (C), whereas the meiocyte of *ms10* ^*35*^ anthers was further diminished (arrow and circle, G). At the tetrad stage, the dyad of T-1082 anthers was divided into tetrads (D), whereas only remnants of the meiocyte remained in *ms10* ^*35*^ anthers (H). Nu, nucleus; Ha, haploid. Bars, 2 μm. (I–Y) Meiotic chromosomes stained with DAPI in T-1082 (I–Q) and *ms10* ^*35*^ (R–Y) at the leptotene (I, R), zygotene (J, S), pachytene (K, L, T, U), diakinesis (M, V), metaphase I (N, W), anaphase I (O, X), telophase I (P, Y), and tetrad (Q) stages. At anaphase I, T-1082 chromosomes were separated to generate two polar sets (O), while *ms10* ^*35*^ chromosomes were aggregated and not segregated clearly (X). At the telophase I stage, T-1082 had generated two different chromosome sets (P), while *ms10* ^*35*^ chromosomes were not segregated clearly and failed to form dyads (Y). At the tetrad stage, T-1082 had generated four tetrahedrally arranged chromosome sets, whereas no tetrad was produced in the *ms10* ^*35*^ mutant. Bars. 10 μm. (This figure is available in colour at *JXB* online.)

PMCs in *ms10* ^*35*^ anthers were degenerated and failed to produce tetrads (Fig. 2). To investigate defects of meiosis in *ms10* ^*35*^, we observed meiocytes using TEM. In T-1082 anthers, uni-nucleate PMCs (Fig. 4A) gave rise to dividing cells (Fig. 4B) and dyads (Fig. 4C), producing tetrads (Fig. 4D) during meiosis. In *ms10* ^*35*^ anthers, PMCs with a well-defined structure were observed as in T-1082 (Fig. 4E). Before long, however, the nuclei of PMCs were crushed without degeneration (Fig. 4F, arrow) and diminished steadily. Eventually only traces remained (Fig. 4G, arrow) and even these disappeared at the end of development (Fig. 4H). No dividing nucleus, dyads, or tetrads were observed in *ms10* ^*35*^ anthers. We further performed chromosome spread experiments using DAPI staining. In T-1082 anthers, chromosomes in PMCs underwent homologous chromosome pairing and synapsis at leptotene/zygotene (Fig. 4I, J) and pachytene (Fig. 4K, L), respectively. Chromosomes were condensed as bivalents at diakinesis and aligned at metaphase I (Fig. 4M, N). The aligned chromosomes then separated and moved towards opposite poles at anaphase I (Fig. 4O), forming dyads at telophase I (Fig. 4P). Tetrads were formed at the end of meiosis as a result of two nuclear segregations (Fig. 4Q). In *ms10* ^*35*^ anthers, meiosis occurred normally until metaphase I (Fig. 4R–W). Chromosomes were abnormally separated at anaphase I (Fig. 4X), failed to form dyads during telophase I (Fig. 4Y), and tetrads were never observed. These results indicated that *ms10* ^*35*^ has a defect in chromosome segregation at anaphase I, resulting in no tetrad formation during meiosis.

### *MS10* ^*35*^ encodes a bHLH transcription factor

**Fig. 5.**
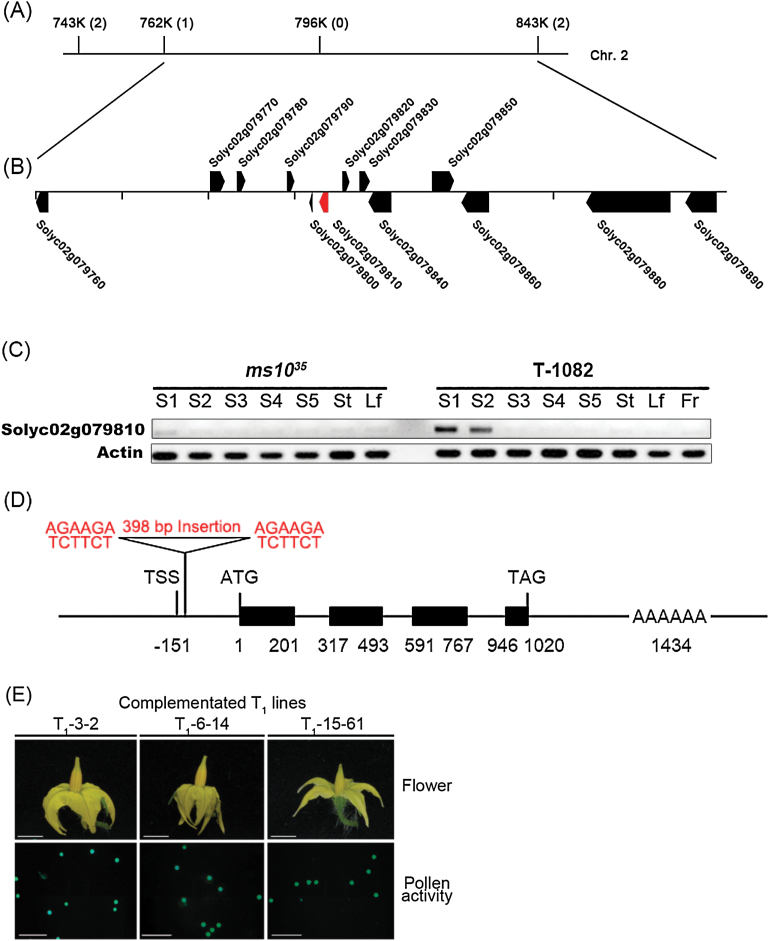
Map-based cloning of the *Ms10* ^*35*^ gene. (A) Fine genetic mapping of the *Ms10* ^*35*^ gene delimited the target gene within an interval between markers 762K and 843K on chromosome 2. Numbers in parentheses indicate the number of recombination events identified between the markers and the target gene. (B) Physical map of the region containing the *Ms10* ^*35*^ gene. Pentagons indicate predicted genes and their directions. The putative *Ms10* ^*35*^ gene (*Solyc02g079810*) is indicated in red. *Solyc02g079760*, bHLH transcription factor; *Solyc02g079770*, DAG protein; *Solyc02g079780*, glycine-rich protein; *Solyc02g079790*, DUF538; *Solyc02g079800*, unknown protein; *Solyc02g079810*, putative *Ms10* ^*35*^ gene; *Solyc02g079820*, pentatricopeptide repeat-containing protein; *Solyc02g079830*, pentatricopeptide repeat-containing protein; *Solyc02g079840*, terpene synthase; *Solyc02g079850*, pleckstrin homology; *Solyc02g079860*, NPH3; *Solyc02g079880*, translation initiation factor; *Solyc02g079890*, terpene synthase. (C) RT-PCR for *Solyc02g079810* from *ms10* ^*35*^ and T-1082 plants. S1–S5, different stages of anther development (S1, meiosis and tetrad stage; S2, young and vacuolated microspore stage; S3, mitosis and maturation stage; S4, dehiscence stage; S5, opened flower stage); St, stem; Lf, leaf; Fr, fruit. *Actin* was used as a loading control. (D) The structure of the *Ms10* ^*35*^ gene encoding a bHLH transcription factor. Black boxes indicate exons. The ATG start site is denoted as position 1 and the other numbers indicate nucleotide distance from the ATG site. A transposon insertion flanked by direct repeat nucleotide sequences (AGAAGA/TCTTCT) was found between the transcription start site (TSS) and the ATG in *ms10* ^*35*^. (E) Flower morphology and pollen viability in *ms10* ^*35*^ transgenic plants complemented with the wild-type *Ms10* ^*35*^ gene. Three independent T_1_ transgenic plants with the *Ms10* ^*35*^ gene showed normal flowers and had viable pollen. Bars, 0.5cm (flower images); 200 μm (pollen viability images). (This figure is available in colour at *JXB* online.)

To examine the inheritance of the *ms10* ^*35*^ gene, F_1_ plants were developed by crossing *ms10* ^*35*^ as a female parent and T-1082 as a male parent (Supplementary Table S2 at *JXB* online). All F_1_ plants developed normal flowers with viable pollen grains. In an F_2_ population generated from F_1_ plants, male-fertile and male-sterile phenotypes segregated in a ratio of approximately 3:1, indicating that the mutation is controlled by a single recessive gene. In previous studies, it was determined that the *ms10* ^*35*^ locus is linked between the *PER-2* and *AA* genes on chromosome 2 (Tanksley and Rick, 1980; Tanksley *et al.*, 1992). Based on this, BAC sequence information from around *PER-2* and *AA* genes was collected, assembled, and used for marker development. Fine mapping of the locus allowed us to position *Ms10* ^*35*^ to within an ~80kb region flanked by markers 762K and 843K (Fig. 5A). A total of 13 hypothetical genes were predicted in this region (Fig. 5B). To explore their transcriptional expression in anthers, RT-PCR was performed. Among them, *Solyc02g079810* was downregulated in *ms10* ^*35*^ anthers compared with T-1082 anthers (Fig. 5C). A sequence similarity search further predicted that *Solyc02g079810* is similar to *Arabidopsis* *DYT1*. Given that *Solyc02g079810* was expressed only in anthers, we considered *Solyc02g079810* as a strong candidate gene for *Ms10* ^*35*^. *Solyc02g079810* contains a 627bp coding sequence comprising four exons and three introns with a 154bp 5′-untranslated region and a 419-bp 3′-untranslated region (Fig. 5D). The transcription start site was located 151bp upstream of the ATG site as confirmed by 5′RACE-PCR analysis. To reveal the structure of the mutation in *ms10* ^*35*^, genome walking was performed. Compared with a T-1082-derived genomic clone containing the *Solyc02g079810* gene, an *ms10* ^*35*^-derived clone had an insertion of a retrotransposable DNA fragment (398bp) in the promoter region near the transcription start site (Fig. 5D and Supplementary Fig. S2 at *JXB* online). These results indicated that very weak expression of the *Ms10* ^*35*^ transcript in *ms10* ^*35*^ anthers resulted from failure of transcription initiation.

To confirm that *Solyc02g079810* was the *Ms10* ^*35*^ gene, we used *Agrobacterium*-mediated transformation to introduce the wild-type *Solyc02g079810* gene, expressed from its native promoter, into plants having the heterozygous genotype (*Ms10* ^*35*^/*ms10* ^*35*^) because the homozygous *ms10* ^*35*^ mutant cannot produce seeds. We generated a total of six T_0_ plants and performed systemic analysis to select T_1_ transgenic plants with the homozygous *ms10* ^*35*^ genotype. We first selected a total of 46 F_1_ plants with the *ms10* ^*35*^/*ms10* ^*35*^ background using *ms10* ^*35*^-specific primers (Supplementary Fig. S3 at *JXB* online) and further screened these plants with *NPTII*-specific and transgene-specific primers, respectively (Supplementary Fig. S3 and Supplementary Table S3 at *JXB* online). Among the T_1_ plants containing the *Ms10* ^*35*^ transgene in the *ms10* ^*35*^/*ms10* ^*35*^ background, eight plants (generated from four individual T_0_ plants) had normal flowers with viable pollen grains (Fig. 5E and Supplementary Table S3) and produced normal fruits with seeds. These results demonstrate that the male sterility of *ms10* ^*35*^ resulted from the loss of function of the *Solyc02g079810* gene.

**Fig. 6.**
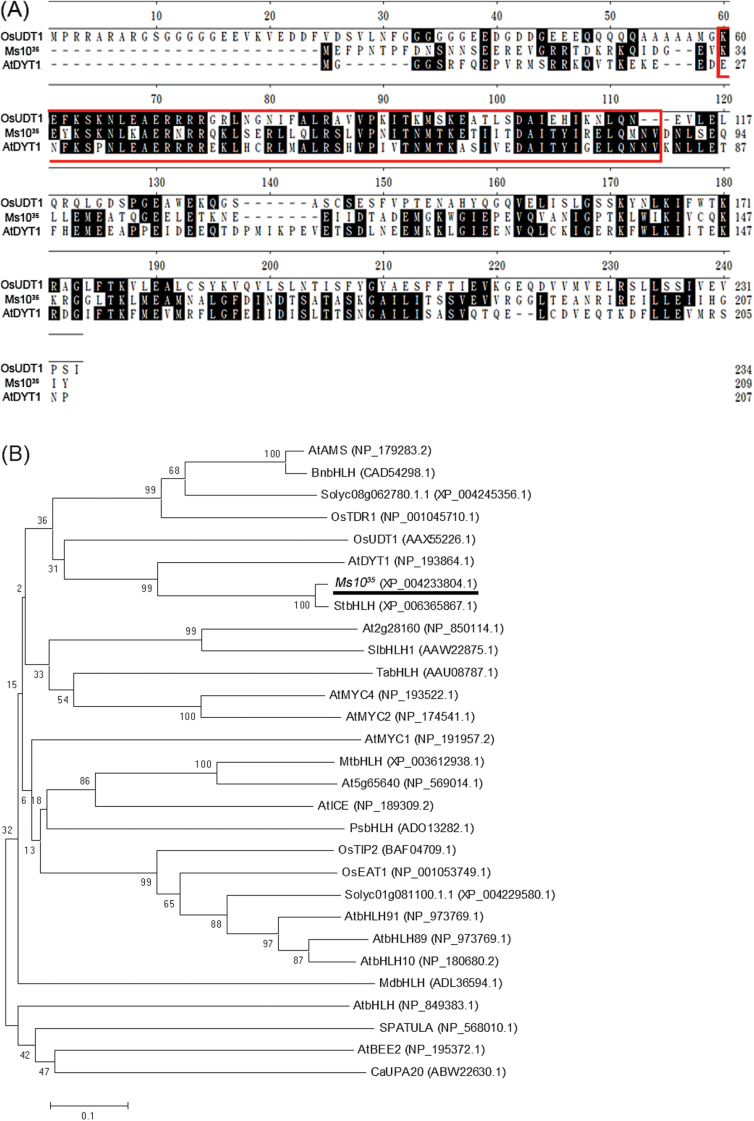
Comparison of Ms10^35^ and its homologues. (A) Amino acid sequence alignment of Ms10^35^ and homologous proteins from potato (StbHLH), *Arabidopsis* (AtDYT1), and rice (OsUDT1). Alignments based on the conserved region of the Ms10^35^ protein were generated using ClustalW of MegAlign (DNASTAR). The conserved bHLH domain is indicated by the outlined box. Black shading indicates identical residues. (B) Phylogenetic tree of Ms10^35^ and other bHLH proteins from several plant species. The phylogenetic tree of deduced amino acid sequences was generated with the neighbour-joining method using MEGA5 software (http://www.megasoftware.net). Bootstrap values (%) are from 1000 replicates, indicated above the nodes. Ms10^35^ is underlined. At, *Arabidopsis thaliana*; Bn, *Brassica napus*; Ca, *Capsicum annuum*; Mt, *Medicago truncatula*; Md, *Malus domestica*; Os, *Oryza sativa*; Ps, *Pisum sativum*; Sl, *Solanum lycopersicum*; St, *Solanum tuberosum*; Ta, *Triticum aestivum*. GenBank accession numbers are given in parentheses. (This figure is available in colour at *JXB* online.)

*Ms10* ^*35*^ encodes a putative transcription factor with a bHLH domain (Fig. 6A). A BLAST search with Ms10^35^ protein sequence showed that the Ms10^35^ protein had the highest similarity to a bHLH protein from *Solanum tuberosum* (91%) and had 47 and 37% similarity to AtDYT1 and OsUDT1, respectively, both of which are required for tapetum development (Jung *et al.*, 2005; Zhang *et al.*, 2006). Amino acid sequence alignment showed that a bHLH domain is highly conserved among these proteins (Fig. 6A). To gain insights into the phylogenetic relationship between Ms10^35^ and other bHLH homologues related to male sterility, phylogenetic analysis was performed. The results showed that Ms10^35^, StbHLH, AtDYT1, and OsUDT1 were within the same clade (Fig. 6B).

### Comparison of global gene expression between *ms10* ^*35*^ and T-1082 anthers by RNA-seq analysis

**Fig. 7.**
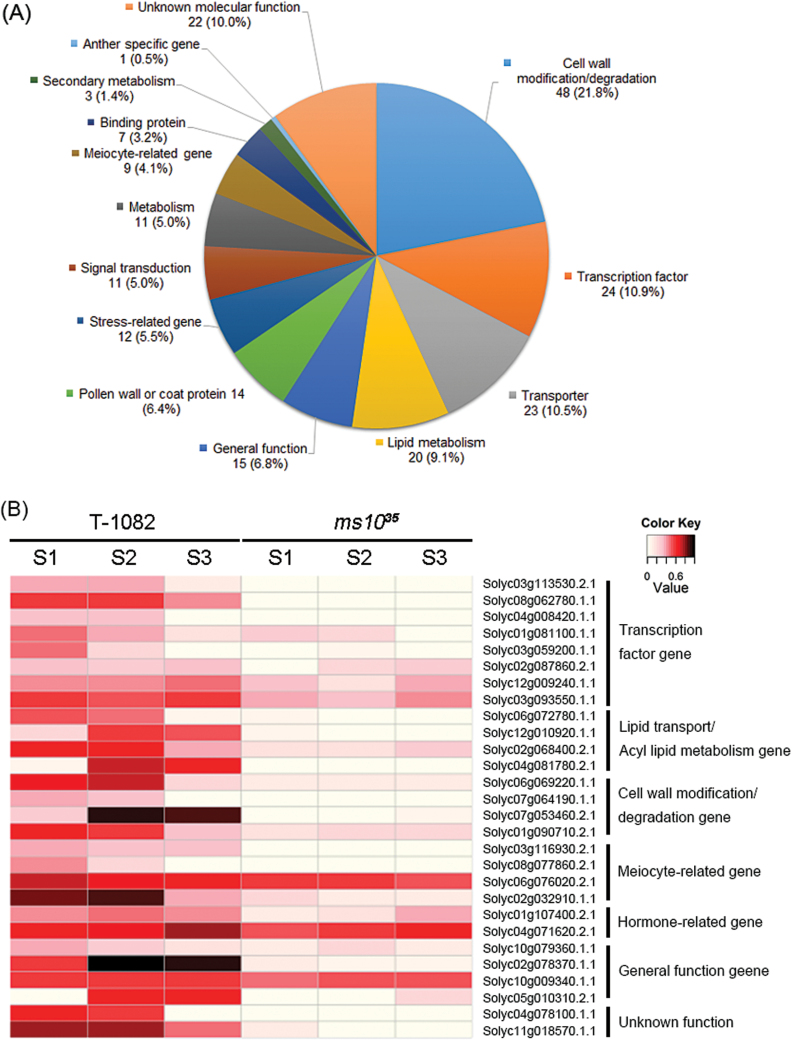
Genes downregulated in the *ms10* ^*35*^ mutant. (A) Classification of genes downregulated in the *ms10* ^*35*^ mutant by gene ontology. Each category of downregulated genes was classified according to putative molecular function and biological process. The number in each category indicates the number of downregulated genes in the *ms10* ^*35*^ mutant and the percentage indicates the number of genes in that category relative to the 220 annotated downregulated genes. (B) Differential expression patterns of representative genes involved in pollen development between T-1082 and *ms10* ^*35*^ anthers. Heat maps show log_2_-scaled reads per kilobase per million reads (RPKM). The intensities of the colours (from 0 to 1: light to dark shading) increase with increasing expression differences as indicated at the upper-right. S1, S2, and S3 indicate different anther developmental stages (S1, meiosis and tetrad stage; S2, young and vacuolated microspore stage; S3, mitosis and maturation stage). Three biological replicates of anthers at each stage were prepared for RNA-seq. (This figure is available in colour at *JXB* online.)

**Fig. 8.**
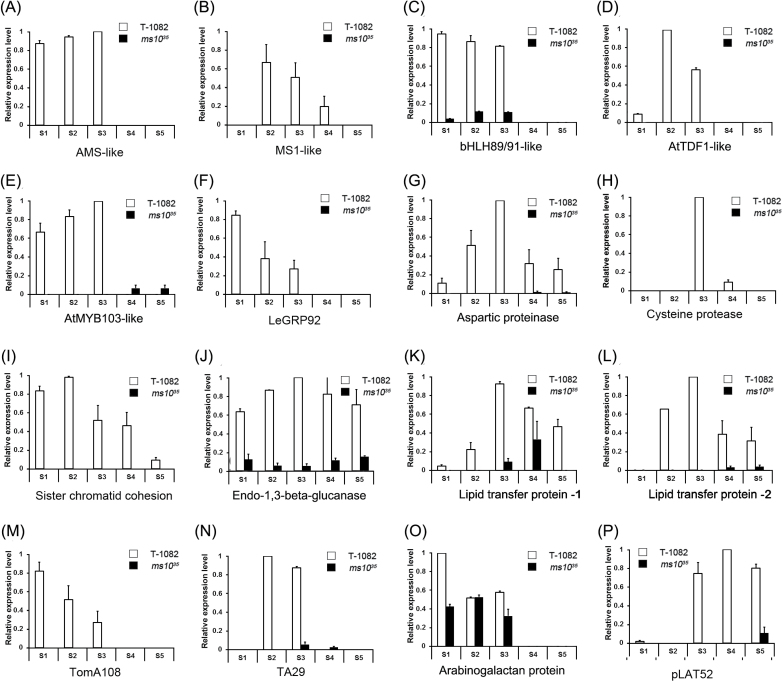
Expression of pollen development marker genes in floral buds of *ms10* ^*35*^ and T-1082 plants. RT-PCR of *AMS-*like (A), *MS1-*like (B), *bHLH89*/*91*-like (C), *AtTDF*-like (D), *AtMYB103*-like (E), *LeGRP92* (F), aspartic protease (G), cysteine protease (H), sister chromatid cohesion (I), endo-1,3-β-glucanase (J), lipid transfer protein (K, L), *TomA108* (M), *TA29* (N), arabinogalactan protein (O), and *pLAT52* (P). The *y*-axis indicates gene expression relative to tomato *Actin*. The results are averages of three independent biological experiments. Error bars show the standard error value of three replicates. RNA samples were collected from floral buds at stages 1–5 of anther development. *AMS-*like, *Solyc08g062780.1.1*; *MS1-*like, *Solyc04g008420.1.1*; *bHLH89*/*91*-like, *Solyc01g081100.1.1*; *AtTDF1*-like, *Solyc03g113530.2.1*; *AtMYB103*-like, *Solyc03g059200.1.1*; *LeGRP92*, *Solyc02g032910.1.1*; aspartic proteinase, *Solyc06g069220.1.1*; cysteine protease, *Solyc07g053460.2.1*; sister chromatid cohesion, *Solyc03g116930.2.1*; endo-1,3-β-glucanase, *Solyc03g046200.1.1*; lipid transfer protein, *Solyc06g059790.2.1* (K) and *Solyc01g095780.2.1* (L); *TomA108*, *Solyc01g009590.2.1*; *TA29*, *Solyc02g078370.1.1*; arabinogalactan protein, *Solyc11g072780.1.1*; *pLAT52*, *Solyc10g007270.2.1*.

To investigate genes regulated by *Ms10* ^*35*^ during pollen development, comparative transcriptome profiling between *ms10* ^*35*^ and T-1082 was performed by RNA-seq. For the identification of distinct genes at three different stages (meiosis/tetrad, young/vacuolated, and mitosis/maturation stages), we analysed all expressed genes using the scatterplot of the DESeq package (Supplementary Fig. S4 at *JXB* online) and selected differentially regulated genes using a false discovery cut-off (5%) and >log_2_ fold difference (*ms10* ^*35*^/T-1082). Finally, 246 genes including 220 genes that were downregulated (Supplementary Table S4 at *JXB* online) and 26 that were upregulated (Supplementary Table S5 at *JXB* online) in *ms10* ^*35*^ relative to T-1082 anthers (*P*<0.05) were discovered by statistical analysis. To investigate further putative functions of downregulated genes in *ms10* ^*35*^, we utilized agriGO (http://bioinfo.cau.edu.cn/agriGO/), the National Center for Biotechnology Information database (http://www.ncbi.nlm.nih.gov), and the genomic database of SGN (http://solgenomics.net). The 220 genes downregulated in *ms10* ^*35*^ were classified into 14 different categories according to molecular function and biological process including transcription factor, cell modification/degeneration, transporter, pollen wall or coat protein, lipid metabolism related, and meiosis related (Fig. 7A and Supplementary Tables S6–S12 at *JXB* online). Furthermore, the 220 genes downregulated in *ms10* ^*35*^ were compared with 435 and 958 downregulated genes in *Arabidopsis dyt1* (Feng *et al.*, 2012) and rice *udt1* (Jung *et al.*, 2005). A total of 65 genes were commonly downregulated across the three organisms. Additional 41 and 15 downregulated genes in *ms10* ^*35*^ were also downregulated in *Arabidopsis dyt1* and rice *udt1*, respectively (Supplementary Tables S4–S12). This result implies that common regulatory machinery is used for pollen development in *Arabidopsis*, rice, and tomato. Some representative genes involved in pollen development and substantially suppressed in *ms10* ^*35*^ are presented in Fig. 7B. To validate the RNA-seq analysis results, we tested the expression patterns of genes known to be involved in pollen development using RT-PCR. The RT-PCR results were consistent with those obtained from RNA-seq data (Fig. 8 and Supplementary Fig. S5). For example, meiosis-related genes such as *Solyc03g116930.2.1* (sister chromatid cohesion), tapetum-specific genes including *Solyc07g053460.2.1* (cysteine protease), and transcription factors such as *Solyc08g062780.1.1* [*ABORTED MICROSPORES*-like (*AMS*-like)] were strongly expressed in T-1082 but considerably downregulated in *ms10* ^*35*^. In addition, *bHLH89*/*91*-like (*Solyc01g081100.1.1*), *AtTDF1*-like (*Solyc03g113530.2.1*), *AtMYB103*-like (*Solyc03g059200.1.1*), aspartic proteinase (*Solyc06g069220.1.1*), endo-1,3-β-glucanase (*Solyc03g046200.1.1*), lipid transfer protein (*Solyc06g059790.2.1* and *Solyc01g095780.2.1*), and arabinogalactan protein (*Solyc11g072780.1.1*) were also downregulated in *ms10* ^*35*^. Transcript levels of two randomly selected genes, *Solyc07g055920.2.1* (*Tomato agamous-like 1*) and *Solyc09g074440.2.1* (*Defenseless1*), which were not differentially regulated in RNA-seq analysis, and those of the control *Actin* gene were similar between *ms10* ^*35*^ and T-1082 (Supplementary Fig. S5).

### *Ms10* ^*35*^ regulates transcription factors involved in anther development

Transcriptional regulation is important for controlling the expression patterns of genes to produce normal pollen (Wilson and Zhang, 2009). Among the 220 downregulated genes in the *ms10* ^*35*^ mutant were 24 transcription factors including bHLH, MYB, NAC, and zinc-finger types (Fig. 7 and Supplementary Table S6). For example, *Solyc03g113530.2.1* and *Solyc08g062780.1.1* are similar to *Arabidopsis DEFECTIVE IN TAPETAL DEVELOPMENT AND FUNCTION1* (*AtTDF1*) and *AtAMS*, which are important for tapetum development and degeneration (Sorensen *et al.*, 2003; Zhu *et al.*, 2008). *Solyc04g008420.1.1* and *Solyc01g081100.1.1* are similar to *Arabidopsis MALE STERILITY1* (*MS1*) and *bHLH91*, respectively, which are involved in tapetum degeneration by regulating PCD-triggered cell death (Vizcay-Barrena and Wilson, 2006; Xu *et al.*, 2010).

### *Ms10* ^*35*^ positively regulates genes for lipid metabolism, cell wall modification/degradation, pollen wall/coat proteins, and transporters

The gene ontology annotation analysis showed that *Ms10* ^*35*^ regulated 20 genes for lipid metabolism, 11 related to energy metabolism, 48 for cell wall modification/degeneration, 14 for pollen wall/coat proteins, and 23 for transporters (Fig. 7 and Supplementary Tables S7–S11). This regulation could be direct or indirect. Among the genes exhibiting altered expression in the *ms10* ^*35*^ mutant, a lipid-related gene, *Solyc03g051960.2.1* (fatty acid CoA reductase) showed high similarity to *Arabidopsis MS2*, which is essential for pollen wall formation (Chen *et al.*, 2011). *Solyc06g072780.1.1* and *Solyc02g068400.2.1* had high similarity to rice *MICROSPORE AND TAPETUM REGULATOR1* (*MTR1*) and *Arabidopsis QUARTET3* (*QRT3*), respectively. *OsMTR1* encodes an arabinogalactan protein known to regulate male sporophytic and reproductive development (Tan *et al.*, 2012). *AtQRT3* encodes a pectin lyase involved in dissolving the PMC callose wall and microspore separation (Preuss *et al.*, 1994). Cell wall modification/degeneration genes such as proteolytic enzyme families induce PCD in tapetum cells (Niu *et al.*, 2013). *Solyc06g069220.1.1* had high similarity to rice *aspartic proteinase 65*, which is involved in tapetum degradation (Huang *et al.*, 2013).

### *Ms10* ^*35*^ regulates genes involved in meiosis

We demonstrated that the *ms10* ^*35*^ mutant was defective in chromosome separation during meiosis (Fig. 4). To identify meiosis-related genes regulated by *Ms10* ^*35*^, we compared downregulated genes in *ms10* ^*35*^ with known *Arabidopsis* meiocyte-specific genes (Yang *et al.*, 2011). We found a total of nine genes related to meiosis (Fig. 7 and Supplementary Table S12). For example, *Solyc03g116930.2.1* was similar to a protein involved in sister chromatid cohesion (Jin *et al.*, 2009). *Solyc08g077860.2.1* encoded a subtilisin-related meiotic serine protease, which is expressed during meiosis and late microsporogenesis in tomato (Riggs *et al.*, 2001). Together, these results imply that *Ms10* ^*35*^ is a master regulator controlling several genes involved in anther development.

## Discussion

### *Ms10* ^*35*^ is important for controlling meiosis and tapetum development

Proper meiosis and development of sporophytic cell layers including the tapetum are essential for successful pollen development in plants (Ma, 2005; Yuan *et al.*, 2009; D. Zhang *et al.*, 2011). In the *ms10* ^*35*^ mutant, duplicated chromosomes were not separated to form dyads (Fig. 4X). This failure of dyad formation at anaphase I resulted in the degradation of meiocytes in *ms10* ^*35*^. Similar defects were found in *Arabidopsis dyt1* and rice *udt1* mutants. In the *dyt1* mutant, meiocytes were not able to complete cytokinesis, resulting in failure of tetrad formation (Zhang *et al.*, 2006; Feng *et al.*, 2012). Transcript analysis showed that expression of the meiosis-specific gene *ROCK-N-ROLLER*/*AtMER3* (*RCK*/*AtMER3*), which is implicated in sister chromatid cohesion, was significantly reduced in *dyt1* (Chen *et al.*, 2005; Zhang *et al.*, 2006), suggesting that *DYT1* regulates the expression of prophase I-related genes. In the rice *udt1* mutant, meiocytes did not produce tetrads due to incomplete meiosis, and *PAIR1* involved in sister chromatid cohesion was downregulated (Jung *et al.*, 2005; Wang *et al.*, 2011). Although these two homologues were not differentially expressed in the RNA-seq analysis, we found that several other meiosis-related genes were downregulated in *ms10* ^*35*^ anthers (Figs 7 and 8). For example, *Solyc03g116930.2.1* is homologous to yeast *PRECOCIOUS DISSOCIATION OF SISTERS PROTEIN5* (*PDS5*), which is important for sister chromatid cohesion such as chromosome condensation, pairing, and synapsis in prophase I (Jin *et al.*, 2009). *Solyc02g032910.1.1* is predicted to encode a protein highly similar to glycine-rich protein, which is important for sporopollenin deposition on meiocyte and exine formation (McNeil and Smith, 2010). Another downregulated gene, *Solyc06g076020.2.1*, is similar to *Heat shock protein 70* (*Hsp70*), which has an important role supporting cyclin-dependent kinase activity in meiosis I in animals (Eddy, 1999). In plants, *Lily messages induced by meiosis 18* (*LIM18*), showing high similarity to eukaryotic *HSP70*, is specifically expressed in microsporocytes during meiosis I (Minami *et al.*, 2000). Our results suggested that the incomplete meiosis of *ms10* ^*35*^ may be due to downregulation of these genes.

Another significant defect in the *ms10* ^*35*^ mutant was abnormal tapetum development. Degeneration of the tapetum and middle cell layer was delayed and, consequently, tapetal cells were greatly expanded and vacuolated (Fig. 3). These phenotypic defects are also commonly observed in *Arabidopsis dyt1* and rice *udt1* mutants (Jung *et al.*, 2005; Zhang *et al.*, 2006). Other mutants impaired in tapetum development such as *Arabidopsis ams* and rice *tapetum degeneration retardation 1* (*tdr1*) also exhibit male sterility (Sorensen *et al.*, 2003; Li *et al.*, 2006). These results support the idea that meiosis and tapetum development are important for pollen development, and that *Ms10* ^*35*^ and its homologues have a conserved role in the completion of meiosis and tapetum development.

RNA *in situ* hybridization analysis revealed that *Ms10* ^*35*^ is expressed exclusively in the meiocyte and tapetal tissues at the early stage of anther development (Supplementary Fig. S6). These transcript results are consistent with the phenotypic defects in meiocytes and tapetal cells of the *ms10* ^*35*^ mutant. To find putative anther-specific *cis*-acting regulatory elements in the *Ms10* ^*35*^ promoter region, we searched the PLACE database (Higo *et al.*, 1999). The *Ms10* ^*35*^ promoter contained several putative transcription binding sites and regulatory sequences. For example, pollen-specific *cis*-elements such as Agamous binding site, Pollen1LeLAT52, and GTGANTG10 were found near the transcription start site (Supplementary Fig. S2), suggesting that these putative *cis*-elements regulate the expression of *Ms10* ^*35*^ in anthers.

### *Ms10* ^*35*^ encodes a bHLH transcription factor

The Ms10^35^ protein sequence had high similarity to StbHLH, AtDYT1, and OsUDT1. Amino acid sequence alignments showed that they contain a conserved bHLH domain at the N-terminal region. The PSORT program (http://mobyle.pasteur.fr/cgi-bin/portal.py?#forms::psort) predicted these proteins to be targeted to the nucleus. Indeed, OsUDT1 is localized to the nucleus and contains a signal peptide at the N terminus (Jung *et al.*, 2005). However, Ms10^35^, StbHLH, and AtDYT do not contain conventional nuclear signal peptides, and whether they are also localized to the nucleus remains to be elucidated. The phylogenetic analysis showed that Ms10^35^, StbHLH, AtDYT1, and OsUDT1 belonged to the same clade. Considering the conserved function of *Ms10* ^*35*^, *AtDYT1*, and *OsUDT1*, it seems possible that *StbHLH* may be involved in meiosis and tapetum development in potato. A clade containing OsTDR1, AtAMS, BnbHLH, and Solyc08g062780.1.1 was very close to the clade containing Ms10^35^. Interestingly, *OsTDR1* and *AtAMS* are regulated by *OsUDT1* and *AtDYT1*, respectively, and are also involved in tapetum degeneration at the post-meiotic stage (Sorensen *et al.*, 2003; Li *et al.*, 2006), suggesting that *Solyc08g062780.1.1* (*AtAMS*-like), which was downregulated in *ms10* ^*35*^, is probably involved in tapetum degeneration. In addition, *OsTIP2* and *OsEAT1* in rice and *AtbHLH89/91* and *AtbHLH10* in *Arabidopsis* are key regulator genes of tapetal PCD (Xu *et al.*, 2010; Niu *et al.*, 2013; Fu *et al.*, 2014). Tomato *Solyc01g081100.1.1* (EAT-like) belongs to the clade including these genes, suggesting that *Solyc01g081100.1.1* could be involved in tapetal PCD. By contrast, genes in other clades were not related to anther development. For example, *AtMYC2* and *AtMYC4* are related to abscisic acid signalling (Abe *et al.*, 2003) and *AtICE* is involved in cold stress (Chinnusamy *et al.*, 2003). These results suggest that the bHLH subfamilies containing *Ms10* ^*35*^ and *AtAMS* have a conserved function and were evolutionarily separated from other bHLH subfamilies.

### Roles of *Ms10* ^*35*^-regulated genes during anther development

**Fig. 9.**
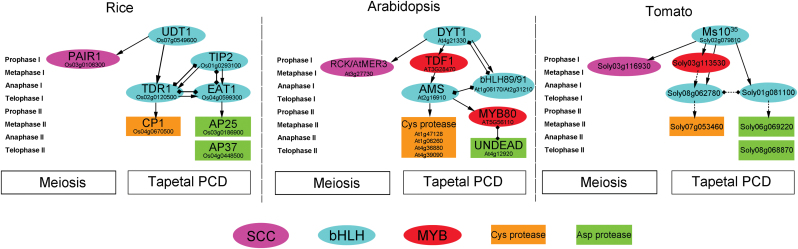
Comparative models for Ms10^35^ and its homologues in *Arabidopsis* and rice that regulate tapetal PCD and meiosis. Arrows represent positive regulation confirmed by RT-PCR or transcriptome analysis. Rhombuses depict protein–protein interaction or protein–promoter interaction confirmed by yeast two-hybrid, quantitative chromatin precipitation-PCR, or electrophoretic mobility shift assay. Dashed lines represent predicted regulation or interaction. The *Arabidopsis* and rice models are based on data reported by Jung *et al.* (2005), Li *et al.* (2006), Zhang *et al.* (2006), Wilson and Zhang (2009), Xu *et al.* (2010), Phan *et al.* (2011), Niu *et al.* (2013), and Fu *et al.* (2014). SCC, sister chromatid cohesion (synaptonemal complexes); bHLH, basic helix–loop–helix transcription factor; MYB, MYB transcription factor; Cys protease, cysteine protease; Asp protease, aspartic proteinase. (This figure is available in colour at *JXB* online.)

A pathway regulated by the *DYT1*–*TDF1*–*AMS*–*bHLH89/91*–*MYB80* transcriptional cascade is suggested to underlie *Arabidopsis* pollen development (Fig. 9). The genes in this pathway are involved in early tapetum function (*DYT1*; Zhang *et al.*, 2006), callose dissolution (*TDF1*; Zhu *et al.*, 2008), and PCD-triggered cell death (*AMS*; Sorensen *et al.*, 2003; Xu *et al.*, 2010). *DYT1* regulates tapetum differentiation during the development of microspore mother cells before meiosis (Zhang *et al.*, 2006). During meiosis, *TDF1* is highly expressed in tapetum cells and meiocytes. *TDF1* functions downstream of *DYT1* and upstream of *AMS* (Zhu *et al.*, 2008). *AMS* is strongly expressed in tapetum cells specifically after meiosis. The *ams* mutant did not successfully undergo PCD, resulting in abnormal tapetal degeneration and retardation (Sorensen *et al.*, 2003; Xu *et al.*, 2010). Rice *TIP2* is associated with anther cell wall specification, tapetal cell size, and PCD by regulating rice *TDR1* and *EAT1* as well as interacting with them (Fu *et al.*, 2014). *EAT1* plays an important role in PCD by regulating proteases and interacting with TIP2 and TDR1 (Niu *et al.*, 2013; Fu *et al.*, 2014). *TDR1* is involved in tapetal cell size and PCD by interacting with TIP2 and EAT1 (Li *et al.*, 2006; Fu *et al.*, 2014), indicating that TDR1–TIP2–EAT1 is formed as a consecutive regulation chain for rice anther development (Fig. 9; Fu *et al.*, 2014). *Arabidopsis bHLH89/91* are homologues of rice *TIP2* and *EAT1*, and interact with AMS, the homologue of rice TDR1 (Xu *et al.*, 2010), suggesting their involvement in tapetal PCD. However, the function of bHLH89/91 is not clear due to a lack of genetic and molecular evidence. *Arabidopsis MYB80* (formerly *MYB103*) encoding a MYB transcription factor is also expressed in the tapetum and microspores (Zhang *et al.*, 2007). The UNDEAD aspartic protease is a direct target of *MYB80*, and the interaction between MYB80 and UNDEAD serves to control induction of tapetal PCD in *Arabidopsis* (Phan *et al.*, 2011). The homologues of these genes have been found in rice (Fig. 9) and have similar functions (Li *et al.*, 2006; Wilson and Zhang, 2009; D. Zhang *et al.*, 2011; X. Zhang *et al.*, 2011; Niu *et al.*, 2013; Fu *et al.*, 2014). In the *ms10* ^*35*^ mutant, *Solyc03g113530.2.1* (*AtTDF*-like), *Solyc08g062780.1.1* (*AtAMS*-like and *OsTDR*-like), and *Solyc01g081100.1.1* (*OsEAT1*-like and *AtbHLH89/91*-like) were significantly downregulated according to the RNA-seq transcriptome and RT-PCR (Fig. 8 and Supplementary Tables S4–S12). These results suggest that this transcriptional cascade for pollen development is well conserved in *Arabidopsis*, rice, and tomato. In addition, the early stage of meiocyte development was controlled by *Ms10* ^*35*^. For example, *Solyc03g116930.2.1*, homologous to yeast sister chromatid cohesion (PDS5), was downregulated in the *ms10* ^*35*^ mutant, which showed abnormal chromosome separation and failure of dyad formation. Similarly, *Arabidopsis RCK* and rice *PAIR1*, which are involved in sister chromatid cohesion, were downregulated in *Arabidopsis dyt1* and rice *udt1* mutants, respectively (Jung *et al.*, 2005; Zhang *et al.*, 2006), implying that *MS10* ^*35*^ and its homologues *AtDYT1* and *OsUDT1* regulate meiosis. Based on these evolutionary relationships and the conserved functions of these proteins, we have presented a model for tomato pollen development (Fig. 9).

In addition to the conserved genes, many genes presumably involved in pollen development in *Arabidopsis* and rice were also downregulated in *ms10* ^*35*^. Cell wall modification and degradation genes such as *Solyc07g064190.1.1* (pectin methylesterase-like), *Solyc06g069220.1.1* (cysteine protease-like), and *Solyc07g053460.2.1* (C1A cysteine proteinase-like) were highly downregulated. Another role of *Ms10* ^*35*^ could be supporting pollen wall deposition by delivering materials and nutrients to developing microspores. For example, orbicules are known to deliver nutrients to sporopollenin of microspores. In T-1082, orbicules were successfully generated along the degenerated tapetum (Fig. 3D). However, in *ms10* ^*35*^, orbicules were not found and no normal sporopollenin structures were observed. In *ms10* ^*35*^, sporopollenin biosynthesis-related genes such as *Solyc12g010920.1.1* (long chain fatty acid reductases) and *Solyc04g081780.2.1* (lipase) were downregulated.

*ms10* ^*35*^ showed a protruded stigma due to significantly reduced anther cone size. *Arabidopsis dyt1* also exhibits a protruded stigma phenotype (Zhang *et al.*, 2006). Tomato *Stigma exsertion* (*Se2.1*) encoding a bHLH transcription factor is the major quantitative trait locus for the development of stamen length (Chen *et al.*, 2007). Interestingly, expression of *Se2.1* (*Solyc02g087860.2.1*) was substantially reduced in *ms10* ^*35*^ anthers, suggesting that *Ms10* ^*35*^ may affect stamen length by regulating *Se2.1*. As we have shown here, many *Ms10* ^*35*^-regulated genes in our RNA-seq data were directly or indirectly involved in anther development. Therefore, our RNA-seq data should provide a good basis for the identification and analysis of new genes involved in anther development in tomato.

## Supplementary data

Supplementary data are available at *JXB* online.

Supplementary Fig. S1. Differences of organ length in *ms10* ^*35*^ and T-1082 flowers.

Supplementary Fig. S2. Nucleotide sequence of the *Ms10* ^*35*^ promoter.

Supplementary Fig. S3. Complementation of *ms10* ^*35*^ transgenic plants with the wild-type *Ms10* ^*35*^ gene.

Supplementary Fig. S4. Scatterplot identification of differentially expressed genes between T-1082 and *ms10* ^*35*^ anthers.

Supplementary Fig. S5. Expression patterns of genes regulated by *Ms10* ^*35*^.

Supplementary Fig. S6. Localization of *Ms10* ^*35*^ expression in T-1082 anthers.

Supplementary Table S1. Primers used in this study.

Supplementary Table S2. Genetic analysis of the *ms10* ^*35*^ gene using an F_2_ population derived from 2–517 (*ms10* ^*35*^) and T-1082 (male fertile) plants.

Supplementary Table S3. Summary of *ms10* ^*35*^ transgenic plants complemented with the wild-type *Ms10* ^*35*^ gene.

Supplementary Table S4. 220 genes downregulated in the *ms10* ^*35*^ mutant.

Supplementary Table S5. 26 genes upregulated in the *ms10* ^*35*^ mutant.

Supplementary Table S6. Transcription factors downregulated in the *ms10* ^*35*^ mutant.

Supplementary Table S7. Lipid metabolism genes downregulated in the *ms10* ^*35*^ mutant.

Supplementary Table S8. Pollen wall or coat protein genes downregulated in the *ms10* ^*35*^ mutant.

Supplementary Table S9. Cell wall modification/degradation genes downregulated in the *ms10* ^*35*^ mutant.

Supplementary Table S10. Transporter genes downregulated in the *ms10* ^*35*^ mutant.

Supplementary Table S11. Energy metabolism-related genes downregulated in the *ms10* ^*35*^ mutant.

Supplementary Table S12. Meiosis-related genes downregulated in the *ms10* ^*35*^ mutant.

Supplementary Data

## Abbreviations:

BAC: bacterial artificial chromosome
bHLH: basic helix–loop–helix
DAPI: 4’,6-diamidino-2-phenylindole
FDA: fluorescein diacetate
HRM: high-resolution-melting
PCD: programmed cell death
PMC: pollen mother cell
RACE: rapid amplification of cDNA ends
RNA-seq: RNA sequencing
RT-PCR: reverse transcription-PCR
SEM: scanning electron microscopy
TEM: transmission electron microscopy.
