# Supplementary material for: Tomato Male sterile 1035 is essential for pollen development and meiosis in anthers
Source: J Exp Bot. 2014 Sep 26;65(22):6693–709. doi: 10.1093/jxb/eru389 (PMC4246194; doi:10.1093/jxb/eru389)
Supplement: Supplementary Data [file supp_65_22_6693__index.html]

Tomato Male sterile 1035 is essential for pollen development and meiosis in anthers — Tomato Male sterile 1035 is essential for pollen development and meiosis in anthers — Supplementary Data 

# Tomato *Male sterile 10**35* is essential for pollen development and meiosis in anthers

## Supplementary Data

Data files

**Files in this Data Supplement:**

- Supplementary Data - Supplementary Data
- Supplementary Data - Supplementary Data
