# Supplementary material for: Tomato Male sterile 1035 is essential for pollen development and meiosis in anthers
Source: J Exp Bot. 2014 Sep 26;65(22):6693–709. doi: 10.1093/jxb/eru389 (PMC4246194; doi:10.1093/jxb/eru389)
Supplement: Supplementary Data [file supp_eru389_jexbot132159_file001.pdf]

**Supplementary material**

# Tomato *Male sterile 10*$^{35}$ is essential for pollen development and meiosis in anthers

**Hee-Jin Jeong, Jin-Ho Kang, Jin-Kyung Kwon, Hak-Sun Choi, Jung Hwan Bae, Hyun-ah Lee, Meiai Zhao, Young-Hee Joung, Doil Choi, Byoung-Cheorl Kang**

## Supplementary method

### RNA *in situ* hybridization

Floral buds of T-1082 plants from pre-meiotic to microspore stages were collected, fixed by vacuum infiltration with FDA buffer (10% formaldehyde, 50% ethanol, and 5% acetic acid), and dehydrated using an ethanol series (30%, 50%, 75%, 90%, and 100%, for 5 min at each step). The full-length *Ms10*$^{35}$ cDNA was labelled with DIG-nick translation mix according to the manufacturer's instructions (Roche, Basel, Switzerland). RNA hybridization and immunological detection of the hybridized probes were performed according to the protocol of Kang *et al.* (2012). Hybridized samples were observed and photographed using a Dimis-M light microscope (Siwon Optical Technology, Anyang, Korea).

**Supplementary Table S1.** Primers used in this study.

| Primer | Purpose | Forward sequence (5'→3') | Reverse sequence (5'→3') |
|---|---|---|---|
| 324K | Fine mapping of *ms10*$^{35}$ | GGAAGTCGAAATCAAGAAAGTAAC | GTTTCAGTATCGCTTGAGAAGC |
| 410K | Fine mapping of *ms10*$^{35}$ | CACTTCAGTTCTCTTCTTTTTTTAA | TCTTTCAATTGAACTCAAAA |
| 556K | Fine mapping of *ms10*$^{35}$ | TGAATGTTTGTCAACAAGTGGG | TATCAGGAAACTTCTTAGAAGCTGG |
| 743K | Fine mapping of *ms10*$^{35}$ | CGACCAATAAGAAAGAGACGTGTC | GCGATTTGTCACATAGAATATACATCTC |
| 762K | Fine mapping of *ms10*$^{35}$ | TCTTACTCACCCTCGACTCTCT | CCAGAGCTCCTAACGTTACTCC |
| 796K | Fine mapping of *ms10*$^{35}$ | GCTAATGATTAATATGTTGAGTGCA | CCCACTTCTATTCTTCTTTCTGTTTG |
| 843K | Fine mapping of *ms10*$^{35}$ | CTACTTATACGAGATGCAAATAGGG | AACGAGCAGGTAATAATGGGT |
| 1142K | Fine mapping of *ms10*$^{35}$ | TATTGATGGAGCGATCGAACTCTTC | ACGGCAGTGTTACGTTAAGTTGGAG |
| 1289K | Fine mapping of *ms10*$^{35}$ | ATTGCAGCGAAATAGGCCAG | ATTGCAATGTCATCACTTAC |
| Solyc02g079760 | RT-PCR for candidate gene | CTTCAGCTTTACTTCAAACTTTATT | AGTAGCATTAAGAGAGTAATTTGGA |
| Solyc02g079770 | RT-PCR for candidate gene | GCTTATTAATCAAATTCTTCGTATC | ATTGATGTACTTATCTCCTCCATAG |
| Solyc02g079780 | RT-PCR for candidate gene | TTAATGTCTCTTATGATTATGATGC | AGTATCATCCAGTTCTTTGGTATAA |
| Solyc02g079790 | RT-PCR for candidate gene | TACACTTTTTCTATCAATTTTCCTC | AAGTAGACTTGTAAGTGTCCATTTT |
| Solyc02g079820 | RT-PCR for candidate gene | ATATTGTCACTTGTAACACGTCATT | ATAGCACTGTAACTGAAATTTATCC |
| Solyc02g079830 | RT-PCR for candidate gene | AATCATACCTGATTTAGTGACTTTC | AATCCTTGTAATCTAATGGTGTAAC |
| Solyc02g079840 | RT-PCR for candidate gene | ACTTGAGCTCTTTACTGATATTGTT | GTGTATATCCTCTTTGATACCACTT |
| Solyc02g079850 | RT-PCR for candidate gene | ACCAGATTCTAGTTATGATTTTGAC | TTCAGAGACTGAATTTCACTTAGTA |
| Solyc02g079880 | RT-PCR for candidate gene | GAACTTCATTACATTTCTAGTCCAT | AATAAAACAGGAATGAAGATATCAC |
| Gene specific primer 1 | Genome walking for *ms10*$^{35}$ | TTTGACGCCTATTTCTCTCAGC | |
| Gene specific primer 2 | Genome walking for *ms10*$^{35}$ | CCTACTTCCCTTTCTTCAGAGTTG | |
| Sister chromatid cohesion | RT-PCR for downstream gene | AGTGAGATCATGAGAATTACAGCTCC | GATGAAGTTTGACAGCACTTTCTTG |
| LeGPR92 | RT-PCR for downstream gene | ATGCAATTAGGAGCCTTGATTC | CAGTTCCAGTTCCTGTTCCG |
| Tom108A | RT-PCR for downstream gene | ATGCAATTAGGAGCCTTGATTC | CAGTTCCAGTTCCTGTTCCG |
| TGAS100 | RT-PCR for downstream gene | TATATAGACATGGCAATGAAATGGC | AGTCAAGACAACGATCAAGAATGC |
| Cysteine protease | RT-PCR for downstream gene | ATTGGTGTCGATTGGAGGAAG | CAAATGCACTTTCCATAAACCC |
| Asparatic protease | RT-PCR for downstream gene | GTGATATTAATTGGCTTCAATGTGAACC | ATACTCGCCGGAACCTGTAACATC |
| TA29 | RT-PCR for downstream gene | AAGATTTTAACCATGAACTTCTTC | ACATTCTTCAGTGTCACATACATC |
| Ms1035 | RT-PCR for downstream gene | AGATCTCTCTGATTCGATTAGCTTCAG | TCTTGAAATGGAAGCAACTCAGG |
| AMS | RT-PCR for downstream gene | TGCAGAGATGTTATGTTTCAGCATC | TCGTCTCTGTCTCTTTCTCCTTCTG |
| MS1 | RT-PCR for downstream gene | TTGTGTCAATGGATCATTGGAAAC | AACCTCTTGCCTAGACACCCATC |
| LeMAN5 | RT-PCR for downstream gene | GCTAGGGTTTTACTTGATGAA | CTTATTTTCTTCCATGTCCTC |
| pLAT52 | RT-PCR for downstream gene | AAGGTGTGACTGATAAAGATGGC | AACCCAACTCATCAAGAGCTTC |
| TAGL1 | RT-PCR for downstream gene | CTTGATGCCAGGGAGTTCAT | ATCGAATTGCTGAGGTGGAG |
| DEF1 | RT-PCR for downstream gene | ATGGGCTATTCAAGAAGGCTAATG | TTCGTAGATTCCTATTCACATCCTTTAG |
| Actin | RT-PCR for downstream gene | GAAATAGCATAAGATGGCAGACG | ATACCCACCATCACACCAGTAT |
| Full length *Ms10*$^{35}$ with *XbaI* | Cloning of *Ms10*$^{35}$ | TCTAGAACGGCGGAGAGATGAAGAAGTAG | AGATCTTTGAGGACGACATTGGTCAAAGAAC |
| Ms-specific primer | Selection for transgenic | TTCTGTTGTGCGGAATTTGAGATAATAC | CCTACTTCCCTTTCTTCAGAGTTG |
| Transgenic specific primer | Selection for transgenic | CCCAGGCTTTACACTTTATG | AGGCCATAGGAGCTTTTACT |
| NPT II | Selection for transgenic | GAAGAACTCGTCAAGAAGGC | GAACAAGATGGATTGCACGC |

**Supplementary Table S2.** Genetic analysis of the *ms10*$^{35}$ gene using an $F_2$ population derived from 2-517 (*ms10*$^{35}$) and T-1082 (male fertile) plants.

| Line | Total | Male fertile (MF) | Male sterile (MS) | Expected ratio (MF:MS) | $X^2$ (*P*-value) |
|---|---|---|---|---|---|
| $P_1$ (2-517) | 9 | 0 | 9 | 0:1 | - |
| $P_2$ (T-1082) | 10 | 10 | 0 | 1:0 | - |
| $F_1$ | 8 | 8 | 0 | 1:0 | - |
| $F_2$ [a] | 236 | 174 | 62 | 3:1 | 0.1968 (0.6573) |

a : Self-pollinated progeny from $F_1$

**Supplementary Table S3.** Summary of *ms10*$^{35}$ transgenic plants complemented with the wild-type *Ms10*$^{35}$ gene.

| | T$_1$ | | | | | | |
|---|---|---|---|---|---|---|---|
| T$_0$ line name [a] | No. of transgenic plants | Genetic background[b] | | Km$^+$ (+)[d] | TSP[e] (+) | Phenotype[f] | |
| | | *Ms10*$^{35}$/*ms 10*$^{35}$ [c] | *ms10*$^{35}$/ *ms10*$^{35}$ | | | Not restored | Restored |
| 3 | 54 | 39 | 15 | 13 | 13 | 9 | 4 |
| 4 | 5 | 3 | 2 | 1 | 1 | 1 | - |
| 6 | 40 | 31 | 9 | 2 | 2 | 1 | 1 |
| 10 | 5 | 2 | 3 | 2 | 2 | 2 | - |
| 13 | 14 | 11 | 3 | 3 | 3 | 2 | 1 |
| 15 | 67 | 53 | 14 | 11 | 11 | 9 | 2 |
| EV[g] | 9 | 6 | 3 | 3 | 3 | 3 | 0 |

a: T$_0$ plants were generated by transforming the wild-type *Ms10*$^{35}$ gene into the heterozygous *Ms10*$^{35}$/*ms10*$^{35}$genetic background produced by crossing *ms10*$^{35}$ X T-1082.

b: genotypes of T1 transgenic plants were confirmed by *ms10*$^{35}$-linked markers and *ms10*$^{35}$-specific marker in Figure S3.

c: *Ms10*$^{35}$/*ms10*$^{35}$ heterozygous or *Ms10*$^{35}$ homozygous genotype.

d: Transgenic plants carrying the kanamycin resistance gene.

e: Transgenic plants with the wild-type *Ms10*$^{35}$ transgene.

f: Restored phenotype confirmed by anther appearance and pollen activity.

g: EV, Transgenic plants carrying empty vector (pCAMBIA2300).

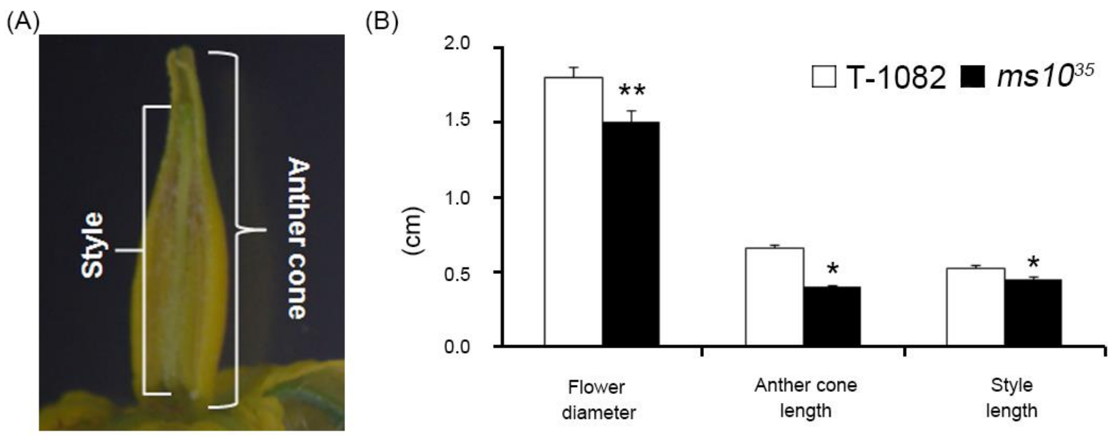


**Supplementary Fig. S1.** Differences of organ length in T-1082 and *ms10*$^{35}$ flowers.
(A) The structure of tomato flower. (B) Flower size and lengths of sexual organs. Flower diameter was measured as the longest distance from the end of a petal to the opposite side of a petal. The style and anther cone were measured from the bottom to top, as shown in (A), in open flowers at stage 5. Each data point represents the mean ± SE of four biological replicates from T-1082 and *ms10*$^{35}$ flowers. Asterisks represent significant differences between T-1082 and *ms10*$^{35}$ plants (unpaired *t* test: * $P < 0.05$; ** $P < 0.01$).

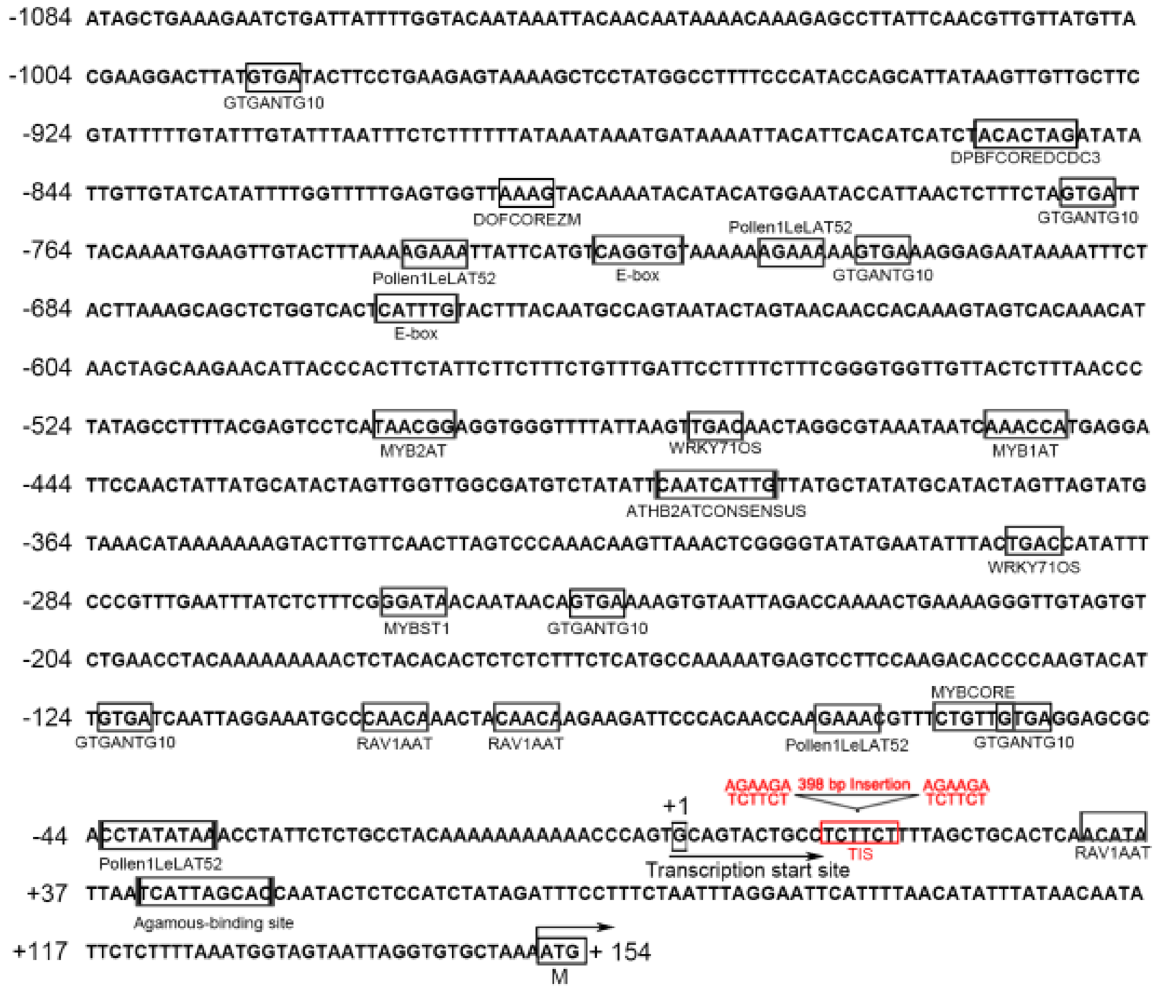

**Supplementary Fig. S2.** Nucleotide sequence of the *Ms10*$^{35}$ promoter.

Numbers to the left of the sequence indicate the position of the nucleotide relative to the deduced transcription start site (+1). The ATG start codon is indicated by M. Putative pollen/anther-specific *cis*-elements are shown in the rectangular boxes. The transposon inserted site in the *ms10*$^{35}$ promoter is shown in the red box. The transposon insertion flanked by direct repeat nucleotide sequences (AGAAGA/TCTTCT) was found just behind the transcription start site in *ms10*$^{35}$ promoter.

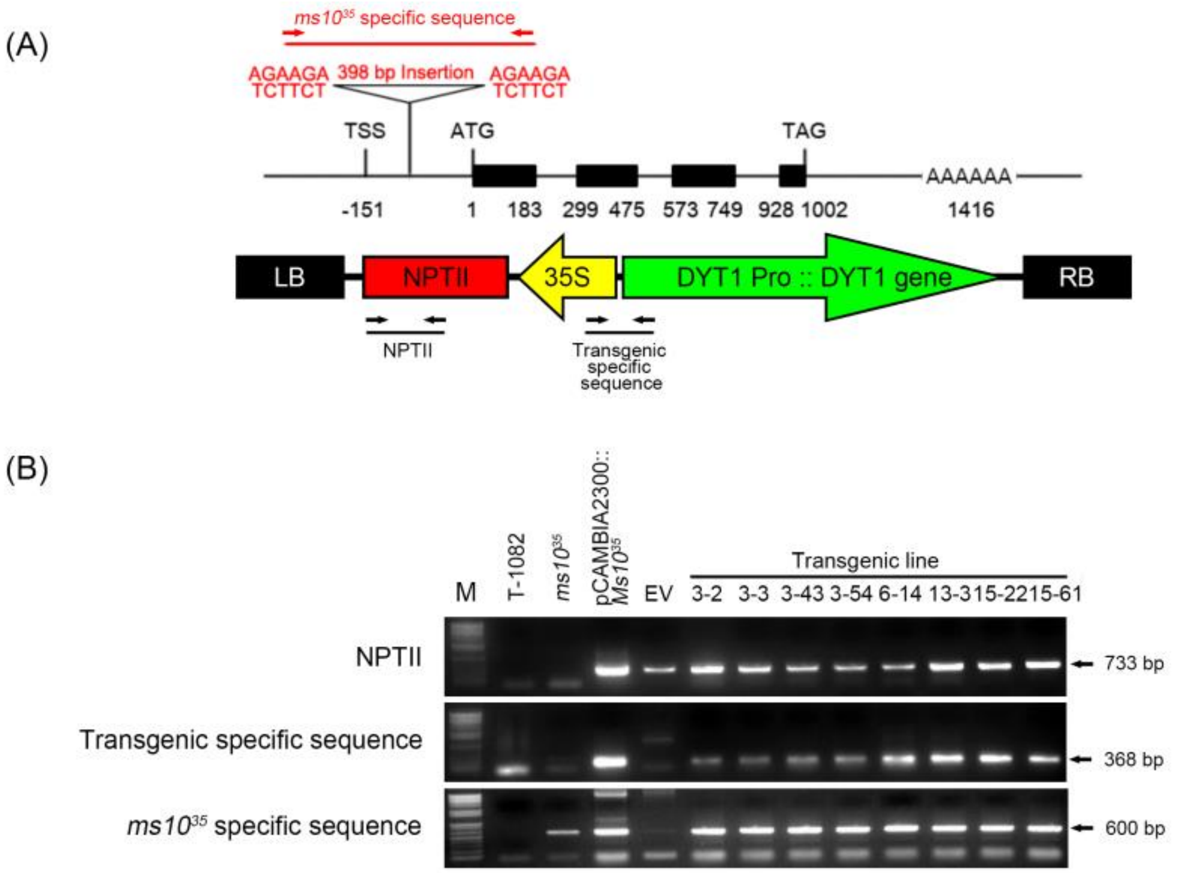


**Supplementary Fig. S3.** Complementation of *ms10*$^{35}$ transgenic plants with the wild-type *Ms10*$^{35}$ gene.

(A) Schematic diagram illustrating the complementation construct and the location of primers used for selection of *ms10*$^{35}$ transgenic plants carrying the *Ms10*$^{35}$ transgene. (B) PCR screening of *ms10*$^{35}$ transgenic plants for those with the *Ms10*$^{35}$ transgene. DNA samples were extracted from each transgenic line and amplified via PCR with primers corresponding to *NPTII*, a *ms10*$^{35}$-specific region, and a transgene-specific region. M: 1-kb plus ladder marker, pCAMBIA2300::*Ms10*$^{35}$: plant transformation vector pCAMBIA2300 carrying *Ms10*$^{35}$ gene, EV: Empty vector (pCAMBIA2300).

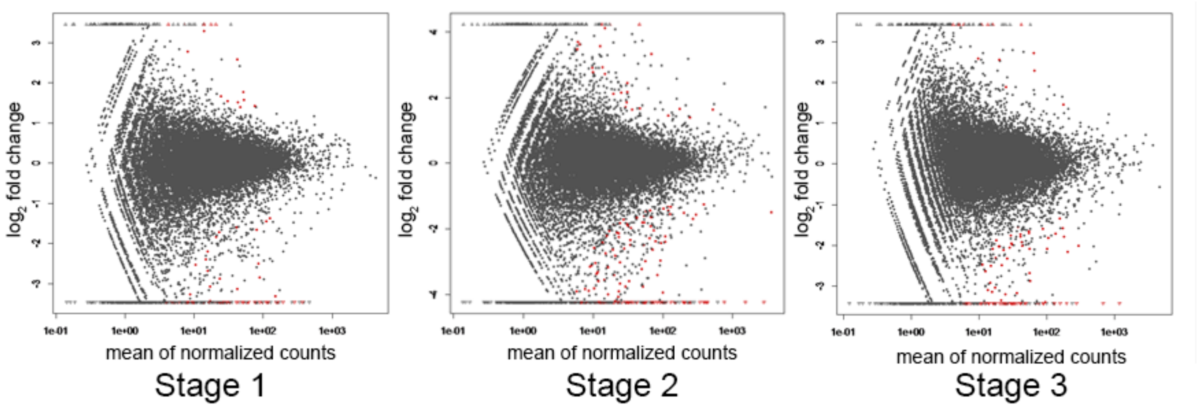


**Supplementary Fig. S4.** Scatterplot identification of differentially expressed genes between T-1082 and *ms10*$^{35}$ anthers.

Plots show the $\log_2$ fold changes against the base means (MA plot) using the DESeq package. Bioconductor packages detected differentially expressed genes between T-1082 and *ms10*$^{35}$ anthers (the red scatter plots). Three biological replicates at each stage from T-1082 and *ms10*$^{35}$ were analyzed with correlation coefficients ($P < 0.05$).

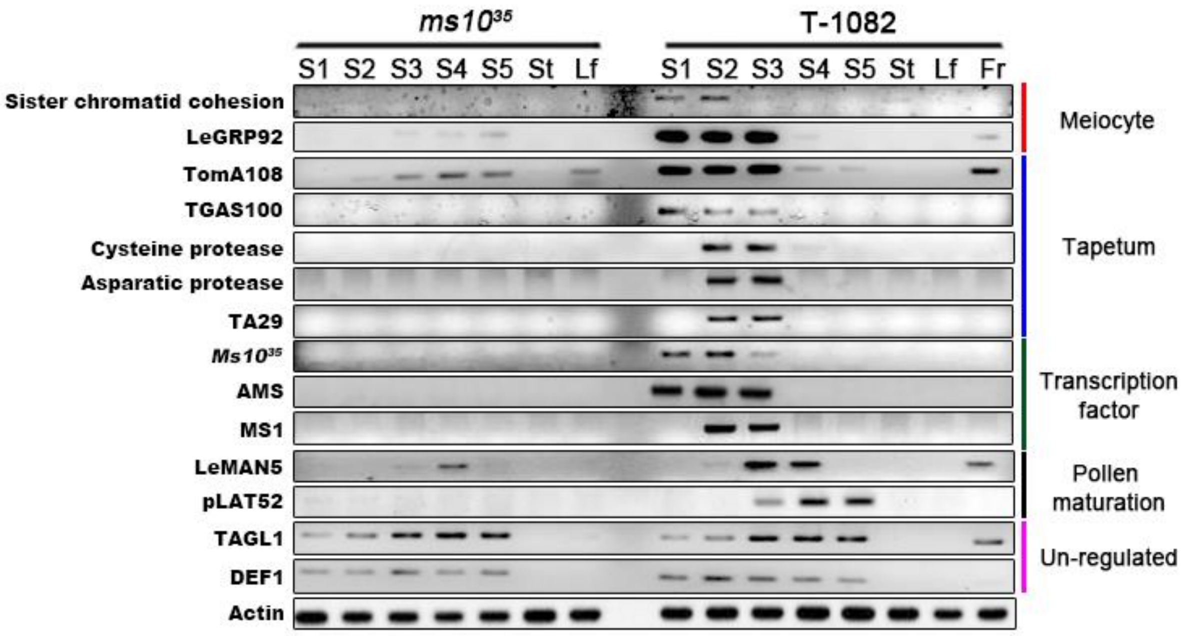


**Supplementary Fig. S5.** Expression patterns of genes regulated by *Ms10*$^{35}$.

PCR results are representative of three biological replicate experiments. *Actin* was used as a control. S1-S5 indicates 5 different stages of anther development (S1: meiosis and tetrad stage, S2: young and vacuolated microspore stage, S3: mitosis and maturation stage, S4: dehiscence stage, S5: opened flower stage). St: stem, Lf: leaf, Fr: fruit. Sister chromatid cohesion: *Solyc03g116930.2.1*, LeGPR92: *Solyc02g032910.1.1*, Tom108A: *Solyc01g009590.2.1*, TGAS100: *Solyc06g064480.2.1*, Cysteine protease: *Solyc07g053460.2.1*, Asparatic protease: *Solyc06g069220.1.1*, TA29: *Solyc02g078370.1.1*, *Ms10*$^{35}$: *Solyc02g079810.1.1*, AMS: *Solyc08g062780.1.1*, LeMAN5: *Solyc06g064520.2.1*, pLAT52, *Solyc10g007270.2.1*; TAGL1, *Solyc07g055920.2.1*; DEF1, *Solyc09g074440.2.1*.

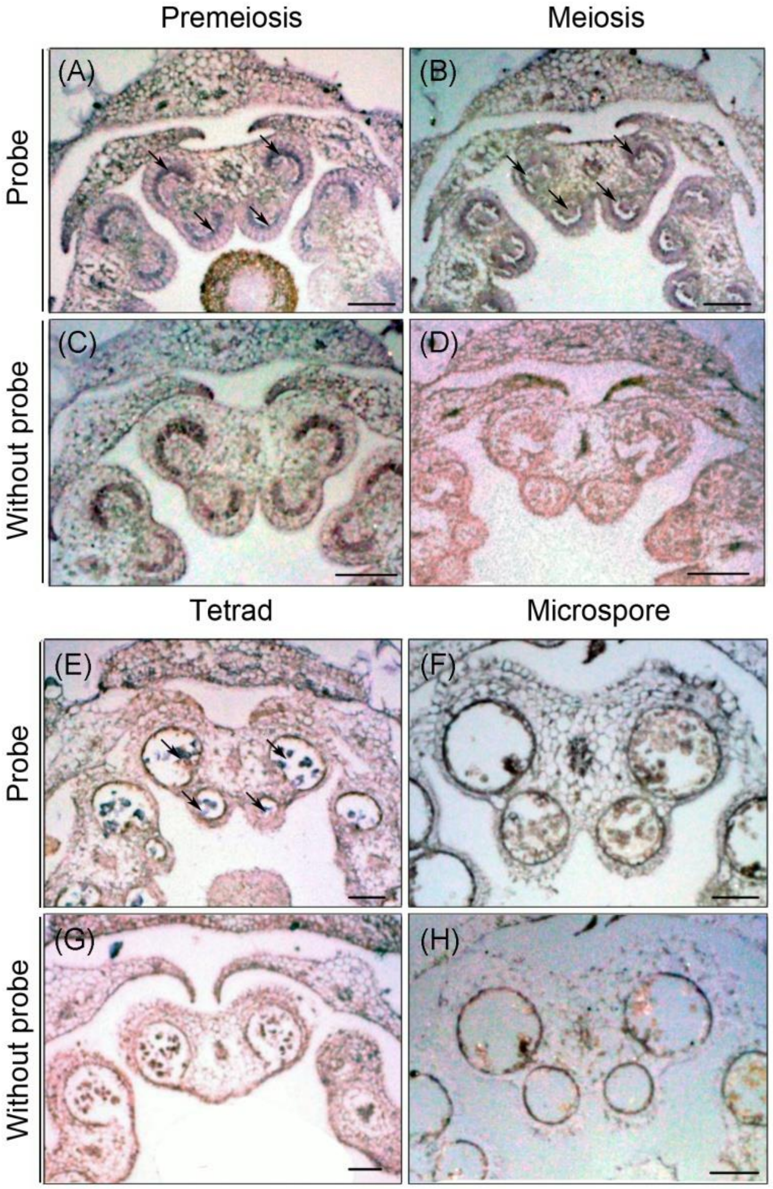


**Supplementary Fig. S6.** Localization of *Ms10*$^{35}$ expression in T-1082 anthers.

RNA *in situ* hybridization was performed on vertical sections of anthers.

(A, B, E, F) *In situ* hybridization with *Ms10*$^{35}$ probe. (A) Pre-meiotic stage. Signals were detected in the meiocyte and tapetal cells (arrow). (B) Meiotic stage. Signals were detected in the meiocyte and tapetal cells (arrow). (E) Tetrad stage. Signals were detected in the meiocyte cells (arrow). (F) Microspore stage. Signals were not detected.

(C, D, G, H) *In situ* hybridization without *Ms10*$^{35}$ probe. No signals were detected at the pre-meiotic (C), meiotic (D), tetrad (G), or microspore (H) stage. Scale bars: 100 μm.
